# Differential healthcare costs in individuals with type 2 diabetes and incident chronic kidney disease in Hong Kong: a latent class trajectory analysis

**DOI:** 10.1007/s00125-026-06698-2

**Published:** 2026-03-18

**Authors:** Yanrong Du, Minglu Zhang, Abby Q. Y. Li, Eric S. H. Lau, Hongjiang Wu, Alice P. S. Kong, Andrea O. Y. Luk, Ronald C. W. Ma, Chun-Kwan O, Lee-Ling Lim, Jenny Y. Z. Zhang, Wai Kit Ming, Weijian Ke, Yanbing Li, Juliana C. N. Chan, Juliana N. M. Lui

**Affiliations:** 1 Department of Medicine and Therapeutics, The Chinese University of Hong Kong, Prince of Wales Hospital, Hong Kong SAR, China; 2 Li Ka Shing Institute of Health Sciences, The Chinese University of Hong Kong, Prince of Wales Hospital, Hong Kong SAR, China; 3 Hong Kong Institute of Diabetes and Obesity, The Chinese University of Hong Kong, Prince of Wales Hospital, Hong Kong SAR, China; 4 Department of Medicine, Faculty of Medicine, University of Malaya, Kuala Lumpur, Malaysia; 5 National Center for Mental Health, Beijing, China; 6 Department of Infectious Diseases and Public Health, Jockey Club College of Veterinary Medicine and Life Sciences, City University of Hong Kong, Hong Kong SAR, China; 7 Institute of Global Governance and Innovation for a Shared Future, City University of Hong Kong, Hong Kong SAR, China; 8 The First Affiliated Hospital of Sun Yat-Sen University, Guangzhou, China

**Keywords:** Chronic kidney disease, Elixhauser Comorbidity Index, EQ-5D-3L, Healthcare costs, Latent class trajectory, Type 2 diabetes, Young-onset diabetes

## Abstract

**Aims/hypothesis:**

Chronic kidney disease (CKD) represents a major and costly comorbidity in type 2 diabetes management. Identifying individuals with high healthcare costs due to CKD will support decision-making for early intervention. We used latent class analysis (LCA) to classify Chinese individuals with type 2 diabetes and incident CKD based on their demographic and clinical profiles.

**Methods:**

For this study, 2886 individuals with type 2 diabetes and incident CKD and complete data for 42 attributes were selected from the prospective Hong Kong Diabetes Register cohort (2007–2019). We used LCA to select 14 variables to classify participants, followed by a hierarchical generalised linear mixed model to evaluate longitudinal healthcare costs among class memberships.

**Results:**

During 109,784 person-years of follow-up, the incidence of CKD was 26.29 per 1000 person-years with a per-patient-per-year (PPPY) cost of US$4395 ± 11,947 (mean ± standard deviation). The four distinct classes used in the LCA based on baseline profiles were as follows: Class 1 (18.3%; PPPY: US$6087 ± 15,519), namely those who were young at onset (44.4 ± 10.3 years), had moderate comorbidities (25.6% had a moderate or high score on the Elixhauser Comorbidity Index [ECI]) and used multiple medications (90.2% used at least three medications); Class 2 (21.2%; PPPY: US$3822 ± 9816), namely those who had old-age onset (66.9 ± 6.9 years), had moderate comorbidities (27.8% had a moderate or high ECI score) and used multiple medications (70.7% used at least three medications); Class 3 (33.9%; PPPY: US$4260 ± 11,725), namely those who were middle-aged at onset (54.2 ± 10.0 years), had few comorbidities (14.0% had a moderate or high ECI score) and used few medications (15.6% used at least three medications); and Class 4 (26.5%; PPPY: US$3923 ± 10,957), namely those who were middle-aged at onset (54.1 ± 7.6 years), had moderate comorbidities (25.3% had a moderate or high ECI score) and used multiple medications (98.9% used at least three medications). Class 1 (young onset) and Class 3 (middle-aged onset) incurred the highest cost during the year of CKD onset, with those in Class 1 having more comorbidities than those in Class 3 at baseline. Multiple healthcare services contributed to the high healthcare costs in Class 1, with costs in Class 3 attributed mainly to post-CKD outpatient and psychiatric care.

**Conclusions/interpretation:**

Those with young-onset type 2 diabetes incurred the highest cost during the year of CKD onset. Individuals with middle-aged onset type 2 diabetes with fewer comorbidities and less intensified treatment at baseline also had subsequent increased healthcare costs.

**Graphical Abstract:**

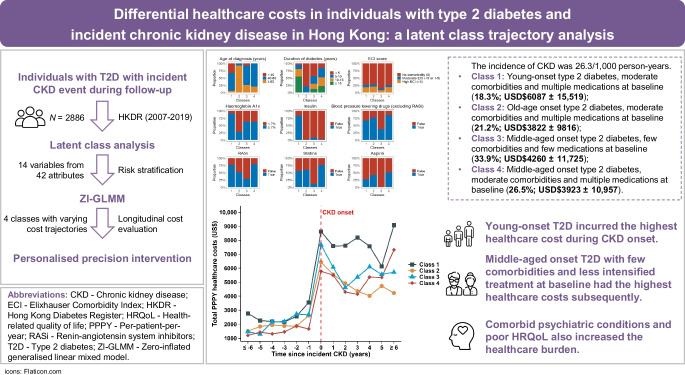

**Supplementary Information:**

The online version of this article (10.1007/s00125-026-06698-2) contains peer-reviewed but unedited supplementary material.


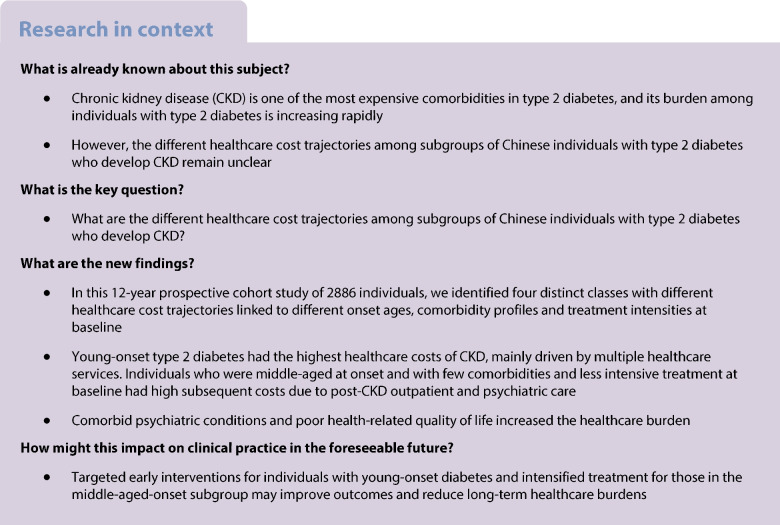


## Introduction

The rising prevalence of type 2 diabetes—the leading cause of chronic kidney disease (CKD), a condition that incurs substantial treatment costs—poses significant public and personal health challenges [1]. The global economic burden of diabetes is projected to rise from US$1.3 trillion in 2015 to US$2.2 trillion in 2030, equivalent to a global gross domestic product of 1.8% and 2.2%, respectively [2]. One in three adults with diabetes may develop CKD during their lifetime [3]. Asians with type 2 diabetes have a higher risk of developing CKD than their European counterparts [4]. In a 33-country survey, 55% of Asians and 40% of Europeans with type 2 diabetes had albuminuria [5].

The management of CKD in type 2 diabetes is complex, incurring both inpatient and outpatient costs. In the USA, the all-cause healthcare costs for individuals with type 2 diabetes and CKD were 121% higher than for individuals with type 2 diabetes only [6]. Progression to end-stage kidney disease (ESKD), often accompanied by multiorgan failure, requires dialysis or kidney transplant for survival. The direct healthcare costs for individuals with CKD vary considerably depending on factors such as CKD stage, comorbidities, medications, procedures, emergency care and hospitalisations [7, 8]. Among Chinese individuals with type 2 diabetes in the Hong Kong Diabetes Register (HKDR), hospitalisation data retrieved from a territory-wide electronic medical record (EMR) system indicated that the incremental costs of CKD were one of the top five most costly complications, with costs of US$14,774 per person in the year of CKD onset and the highest residual costs in subsequent years [9].

Multidisciplinary care with early diagnosis, the control of multiple risk factors and the use of organ-protective drugs would delay CKD progression and ESKD onset and reduce long-term healthcare costs [10]. However, due to the asymptomatic nature of CKD [1] and low public awareness, many individuals with CKD are undiagnosed, untreated or suboptimally managed. In China, only 10% of individuals with CKD are aware of their condition [11], calling for proactive screening and timely intervention to improve outcomes and health-related quality of life (HRQoL) [12].

Type 2 diabetes and CKD are heterogeneous in their causes, trajectories and consequences. Identifying subgroups of individuals with type 2 diabetes who have different baseline profiles associated with varying trajectories of healthcare costs will improve risk stratification and the precision of intervention [13], although such data are scarce. This study employed latent class analysis (LCA) with demographic, clinical and psychosocial-behavioural indicators to unravel the heterogeneity of CKD-associated costs and future healthcare costs due to CKD onset.

## Methods

### Study design and data collection

This 12-year prospective cohort study enrolled 24,058 individuals with type 2 diabetes in the HKDR between 2007 and 2019. Recruitment, definitions and biochemical investigations are detailed elsewhere [14]. The original HKDR cohort is predominantly Chinese, aged 10–96 years, with a balanced sex distribution and wide variation in occupation and education, broadly representing the population with type 2 diabetes in Hong Kong. Established in 1995 at the Prince of Wales Hospital, Hong Kong, the HKDR is a research-driven quality improvement initiative in which regular comprehensive assessments are undertaken to stratify risk and promote shared decision-making. Using the unique identity number within the registry, we regularly retrieved clinical outcomes from the territory-wide EMR system of the Hong Kong Hospital Authority (HA) to identify care gaps. The study adhered to the Declaration of Helsinki, and all participants provided written consent. The study was approved by the Clinical Research Ethics Committee of The Chinese University of Hong Kong (reference no. 2009.421).

### Baseline clinical assessment

All participants underwent structured comprehensive assessments every 2 years by nurses, when sociodemographic and lifestyle factors, laboratory investigations, medication use and past medical history were documented. Sociodemographic factors included sex, age at assessment, age at diagnosis, diabetes duration, education, occupation and family history of diabetes. Lifestyle factors included smoking, alcohol, exercise frequency, adherence to a balanced diet in the past 3 months and self-monitoring of blood glucose. The EuroQol five-dimensional three-level questionnaire (EQ-5D-3L) was used to assess HRQoL. Other parameters included waist circumference, height, weight, systolic and diastolic blood pressure (BP). Fasting blood and random urine samples were analysed for haemoglobin A_1c_ (HbA_1c_), blood glucose and lipids, and kidney function (estimated glomerular filtration rate [eGFR] and urinary albumin/creatinine ratio [UACR]). Medical treatments were classified into glucose-, BP- and lipid-lowering drugs, plus aspirin.

The HA accounts for 90–95% of inpatient and 30% of outpatient services including primary care. Due to the heavily subsidised drug costs in the public setting, the majority of patients requiring long-term medications are managed in hospital- or community-based clinics operated by the HA with a single territory-wide EMR system. We used the EMR system to retrieve data on diabetes-related complications, including severe hypoglycaemia requiring hospitalisation, cardiovascular disease (CVD, including myocardial infarction, coronary heart disease, stroke and peripheral vascular disease), ESKD and any-site cancers, identified using the International Classification of Disease, Ninth Revision, codes for all admissions and procedures (electronic supplementary material [ESM] Table 1). The burden of comorbidities at baseline was evaluated using the van Walraven weighting algorithm for the Elixhauser Comorbidity Index (ECI). Full covariate details are provided in ESM Table 2.

### Inclusion and exclusion criteria

This study included Chinese individuals with type 2 diabetes aged ≥18 years enrolled in the HKDR since 23 November 2007 with incident CKD up to the censor date of 31 December 2019. All participants were free of CKD at enrolment. Exclusion criteria included: (1) type 1 diabetes defined as ketotic presentation or continuous requirement of insulin within 12 months of diagnosis; (2) unknown type of diabetes; (3) self-reported non-Chinese or unknown nationality; (4) aged <18 years; (5) incomplete data on healthcare cost or no EQ-5D-3L; and (6) no incident CKD by the censor date.

### Definition of incident CKD

CKD was defined as an eGFR <60 ml/min per 1.73 m^2^ estimated by the CKD Epidemiology Collaboration equation on at least two occasions 90–365 days apart. Incident CKD was defined as the first occurrence of CKD during follow-up in participants who had no CKD at baseline (eGFR ≥60 ml/min per 1.73 m^2^).

### Measurement of healthcare costs

Annual total healthcare costs were assessed on a per-patient-per-year (PPPY) basis from enrolment to the censor date. Hospitalisation costs were estimated by multiplying the length of stay by the daily cost of the type of healthcare services used. These included accident and emergency (A&E) care, general and specialist outpatient departments (OPDs), day centres for procedures, admissions to acute general wards, high-dependency units (HDUs), intensive care units (ICUs), psychiatric wards and facilities for convalescence, rehabilitation or infirmary care. Unit costs for these services were based on charges applied to non-eligible individuals published in the 2023 Hong Kong Gazette and HA Ordinance (ESM Table 3). The costs were specific to the year of analysis, avoiding inflation adjustments. Sensitivity analyses used winsorisation for all costs at the 0.1% threshold to mitigate the impact of extreme outliers on the cost estimates. The PPPY costs were categorised relative to the year of occurrence of CKD, indicated by year 0, with negative and positive values representing years preceding and following CKD onset, respectively.

### Latent Class Analysis (LCA)

Based on a literature review and input from clinical experts, 14 indicator variables were used (ESM Table 4), including age at diagnosis, diabetes duration, lifestyle factors, the severity of ECI comorbidities, HbA_1c_, UACR and medication use before incident CKD. Given the categorical nature of the indicators, we employed a standard latent class model without covariates [15, 16], which specifies the probabilities of observed indicators for given latent classes and estimates parameters via maximum likelihood estimation. For each individual, posterior class membership probabilities were computed from the observed data and the estimated parameters. Individuals were assigned to the class with the maximum posterior probability [17].

Models with two to six latent classes were fitted. Model fit and interpretability were assessed using information criteria and clinical plausibility. The Bayesian information criterion (BIC) showed the strongest performance and was used to select the optimal model. The model with the lowest BIC was selected.

Multinomial logistic regression (MNL) was used to estimate the effect of covariates on latent class membership [16], with Class 2 as the reference. All baseline variables were included as covariates except the 14 LCA indicators. To account for uncertainty in class assignment, we incorporated observation-specific weights derived from the individual posterior probabilities into the MNL models, yielding adjusted odds ratios (aORs) and 95% confidence intervals (CIs) for covariate associations with class membership.

### Statistical analysis

We analysed the LCA classes on longitudinal healthcare costs using a hierarchical zero-inflated generalised linear mixed model (ZI-GLMM) with log-link function and gamma distribution to accommodate non-negative, right-skewed data with high zero-inflation (>10%) [18, 19]. With a log-link function, the variables are associated with a proportional change in the mean total costs. A random intercept and a random slope for the year were specified at the individual level to allow time effects to vary across participants. This framework integrates generalised linear modelling, random effects and zero-inflation to address non-independence of observations as well as skewness and excess zeros [20]. We excluded variables in the LCA and adjusted the relationship for alcohol use, family history of diabetes, diastolic BP, plasma albumin, alkaline phosphatase, eGFR, prior severe hypoglycaemia, prior CVD, prior cancer and EQ-5D-3L. As part of sensitivity analyses, we also conducted hierarchical linear modelling (HLM) to evaluate the robustness of the findings [21]. Linear regression is widely used in cost analyses due to its interpretability and, with a sufficiently large sample size, often yields results consistent with alternative models [19]. Throughout model development, model selection favoured lower Akaike’s information criterion and BIC values.

Baseline missingness ranged from 0% to 7.1% (ESM Table 5). Little’s missing completely at random (MCAR) test indicated data were missing completely at random (χ^2^=19,807, *df*=21,324, *p*>0.05). No significant differences were observed in key baseline characteristics between participants with and without missing data. Thus, multiple imputations with chained equations under a missing-at-random assumption were used to generate ten imputed datasets with 50 iterations, with convergence assessed visually (ESM Fig. 1) [22]. Estimates from imputed datasets were combined using Rubin’s rules [23].

For baseline characteristics, continuous variables were reported as the median (interquartile range [IQR]) and mean (standard deviation [SD]), and categorical variables were reported as frequencies (percentages). The frequencies of diabetes-related complications and PPPY costs were described by class. Locally estimated scatterplot smoothing (LOESS) was used to explore healthcare cost trajectories over time by class. A *p* value of <0.05 was considered significant. All analyses were performed in R (version 4.4.1; https://www.r-project.org/).

## Results

### Baseline characteristics of the cohort

From the cohort of 15,974 individuals (2007–2019) with a median (IQR) follow-up of 7.3 (4.6, 9.2) years (109,784 person-years) after excluding missing variables, 2886 individuals with type 2 diabetes experienced their first CKD event with an incidence rate of 26.29 per 1000 person-years (ESM Fig. 2). In this cohort (43.7% women), the mean (SD) age was 63.9 (10.0) years, age at type 2 diabetes diagnosis was 55.1 (11.4) years and diabetes duration was 8.7 (7.7) years, while 55.3% reported a family history of diabetes. At baseline, 60.3% had an HbA_1c_ level ≥7% (53 mmol/mol), 91.2% were treated with glucose-lowering drugs, 64.0% were treated with BP-lowering drugs, 52% (47% on statins) were treated with lipid-lowering drugs and 78.0% had no comorbidities (Table 1). ESM Table 6 compares the baseline characteristics of included and excluded individuals. Among the total 2886 study participants, only 3 (0.1%) were treated with sodium-glucose transporter 2 inhibitors and 2 (0.1%) were treated with glucagon-like peptide-1 receptor agonists at baseline. These drugs were introduced in the HA formulary in 2015 and 2008, respectively, when evidence for their organ-protective effects was just emerging [24]. Individuals with pre-existing CKD (*n*=2708) were older and had worse renal function and a greater comorbidity burden. They showed a higher prevalence of diabetes-related complications (hypoglycaemia, CVD and ESKD) and higher all-cause mortality with markedly higher total healthcare costs for the majority of hospital service types than the group with incident CKD (ESM Table 7).

**Table 1:** Baseline characteristics of 2886 participants with type 2 diabetes and incident CKD

Variable	Overall (*N*=2886)
Personal characteristics	
Follow-up, years, median (IQR)	3.6 (1.5, 5.9)
Sex, *n* (%)	
Female	1260 (43.7)
Male	1626 (56.3)
Age at assessment, years, mean (SD)	63.9 (10.0)
Age at assessment, years, *n* (%)	
<60	962 (33.3)
≥60	1924 (66.7)
Age at diagnosis, years, mean (SD)	55.1 (11.4)
Age at diagnosis, years, *n* (%)	
<40	257 (8.9)
40–60	1628 (56.4)
≥60	1001 (34.7)
Duration of diabetes, years, mean (SD)	8.7 (7.7)
Duration of diabetes, years, *n* (%)	
<5	1065 (36.9)
5–10	694 (24.0)
10–15	544 (18.8)
≥15	584 (20.2)
Education level, *n* (%)	
Primary, illiterate or other	1459 (50.5)
Middle or high school	1165 (40.4)
College or above	262 (9.1)
Occupation status, *n* (%)	
Employed (full-time/part-time)	717 (24.8)
Housewife/student/unemployed	914 (31.7)
Retired	1255 (43.5)
Family history of diabetes, *n* (%)	
False	1290 (44.7)
True	1596 (55.3)
Lifestyle factors	
Consumption of alcohol, *n* (%)	
Never	1674 (58.0)
Ex-drinker	417 (14.5)
Current drinker	795 (27.6)
Smoking, *n* (%)	
Never	1881 (65.2)
Ex-smoker	676 (23.4)
Current smoker	329 (11.4)
Frequency of physical activity, *n* (%)	
No regular activity	1111 (38.5)
Less than three times/week	329 (11.4)
Three or four times/week	241 (8.3)
Five times/week	148 (5.1)
More than five times/week	1057 (36.6)
Adherence to balanced diet in last 3 months, *n* (%)	
No	336 (11.7)
Occasional	992 (34.4)
Yes	1558 (54.0)
Self-monitoring of blood glucose, *n* (%)	1919 (66.5)
Clinical and biochemical characteristics	
Central obesity, *n* (%)	1657 (57.4)
Body mass index, kg/m^2^, *n* (%)	
<25	1304 (45.2)
25–30	1129 (39.1)
≥30	452 (15.7)
HbA_1c_, *n* (%)	
<7% (<53 mmol/mol)	1146 (39.7)
≥7% (≥53 mmol/mol)	1740 (60.3)
Fasting plasma glucose, mmol/l, *n* (%)	
<7	1159 (40.2)
≥7	1727 (59.8)
Diastolic BP, mmHg, *n* (%)	
<75	1041 (36.1)
75–85	1007 (34.9)
≥85	837 (29.0)
Systolic BP, mmHg, *n* (%)	
<125	616 (21.4)
125–140	899 (31.2)
≥140	1370 (47.5)
Total cholesterol, mmol/l, *n* (%)	
<5.2	2111 (73.2)
≥5.2	775 (26.9)
Triglycerides, mmol/l, *n* (%)	
<1.69	1782 (61.7)
1.69–2.26	515 (17.9)
≥2.26	589 (20.4)
High-density lipoprotein cholesterol, mmol/l, *n* (%)	
<1.3	1501 (52.0)
1.3–1.55	833 (28.9)
≥1.55	552 (19.1)
Low-density lipoprotein cholesterol, mmol/l, *n* (%)	
<1.4	178 (6.2)
1.4–1.8	355 (12.3)
1.8–2.6	1029 (35.6)
≥2.6	1324 (45.9)
Haemoglobin, g/l, *n* (%)	
Normal	2215 (76.7)
Anaemia	671 (23.3)
Plasma albumin, g/l, *n* (%)	
<35	38 (1.3)
35–50	2806 (97.2)
≥50	42 (1.5)
Alanine aminotransferase, mmol/l, *n* (%)	
<10	72 (2.5)
10–40	2390 (82.8)
≥40	424 (14.7)
Alkaline phosphatase, mmol/l, *n* (%)	
<44	164 (5.7)
44–147	2681 (92.9)
≥147	41 (1.4)
Bilirubin, µmol/l, *n* (%)	
<5	68 (2.4)
5–21	2678 (92.8)
≥21	140 (4.8)
eGFR, ml/min per 1.73 m^2^, *n* (%)	
60–90	2365 (81.9)
≥90	521 (18.1)
UACR, mg/mmol, *n* (%)	
<3	1347 (46.7)
3–30	1025 (35.5)
≥30	514 (17.8)
Medical treatments	
Glucose-lowering drugs, *n* (%)	2633 (91.2)
Insulin, *n* (%)	537 (18.6)
BP-lowering drugs (excluding RASi), *n* (%)	1846 (64.0)
RASi (including ACEi and ARB), *n* (%)	1602 (55.5)
ACEi, *n* (%)	1244 (43.1)
ARB, *n* (%)	365 (12.7)
Lipid-lowering drugs (excluding statins), *n* (%)	142 (4.9)
Statins, *n* (%)	1356 (47.0)
Aspirin, *n* (%)	783 (27.1)
Medical history	
Sensory neuropathy, *n* (%)	194 (6.7)
Diabetic retinopathy, *n* (%)	992 (34.4)
Prior severe hypoglycaemia, *n* (%)	302 (10.5)
Prior CVD, *n* (%)	819 (28.4)
Prior cancer, *n* (%)	208 (7.2)
ECI score (range: <0–30), *n* (%)	
No comorbidity (0)	2250 (78.0)
Moderate (<0 or 1–5)	198 (6.9)
High (>5)	438 (15.2)
EQ-5D-3L	
Mobility, *n* (%)	
No problems	2628 (91.1)
Some or severe problems	258 (8.9)
Self-care, *n* (%)	
No problems	2805 (97.2)
Some or severe problems	81 (2.8)
Usual activities, *n* (%)	
No problems	2711 (93.9)
Some or severe problems	175 (6.1)
Pain/discomfort, *n* (%)	
No problems	2108 (73.0)
Some or severe problems	778 (27.0)
Anxiety/depression, *n* (%)	
No problems	2323 (80.5)
Some or severe problems	563 (19.5)

Due to imputation and the application of Rubin's rules, the frequencies of categorical variables may not sum up to the overall total, and the corresponding percentages may not precisely add up to 100%

ACEi, angiotensin-converting enzyme inhibitor; ARB, angiotensin receptor blocker; BMI, body mass index; eGFR, estimated glomerular filtration rate; EQ−5D−3L, EuroQol five dimensional three-level questionnaire; ECI, Elixhauser comorbidity index; IQR, interquartile range; RASi, renin–angiotensin system inhibitors; SD, standard deviation; UACR, urine albumin/creatinine ratio

### Baseline characteristics of the latent classes

The four-class model had the best statistical fit (ESM Table 8) with clinically distinct classes based on baseline characteristics at enrolment: Class 1 (18.3%) had young-onset type 2 diabetes (YOD) (mean [SD]: 44.4 [10.3] years), had moderate comorbidities (25.6% had a moderate or high ECI score) and used multiple medications (90.2% used at least three medications); Class 2 (21.2%; reference latent class) had old-age onset (mean [SD]: 66.9 [6.9] years), had moderate comorbidities (27.8% had a moderate or high ECI score) and used multiple medications (70.7% used at least three medications); Class 3 (33.9%) had middle-aged onset (mean [SD]: 54.2 [10.0] years), had few comorbidities (14.0% had a moderate or high ECI score) and used few medications (15.6% used at least three medications); and Class 4 (26.5%) had middle-aged onset (mean [SD]: 54.1 [7.6] years), had moderate comorbidities (25.3% had a moderate or high ECI score) and used multiple medications (98.9% used at least three medications). The definitions used for naming these classes are summarised in ESM Table 9.

ESM Fig. 3 shows the distribution of 14 indicators in each of the four classes. Among the four classes, Class 1 had the longest diabetes duration (mean [SD]: 16.4 [8.2] years), whereas Class 2 had the shortest (2.8 [2.7] years). Class 1 had the highest proportion (90.6%) of individuals with a HbA_1c_ level of ≥7% (53 mmol/mol) and Class 2 had the lowest proportion (27.8%). Class 1 had the highest usage of insulin (83.2%).

Covariates associated with class membership identified via MNL relative to Class 2 are shown in ESM Table 10. Class 2 was used as the reference, as it had the lowest total PPPY costs. Compared with individuals aged <60 years, those aged ≥60 years were less likely to be classified as Classes 1, 3 or 4 than as Class 2 (aOR: 0.07–0.12, all *p*<0.001). Compared with non-users, users of angiotensin-converting enzyme inhibitors (ACEis) or angiotensin II receptor blockers (ARBs) had higher odds of being classified as Classes 1 or 4 (aOR: 2.70–3.60, all *p*<0.01) but lower odds of being classified as Class 3 (aOR: 0.26–0.41, all *p*<0.001) than as Class 2. Compared with individuals without prior CVD, those with prior CVD had lower odds of being classified as Classes 1 or 3 (aOR: 0.11–0.49, all *p*<0.01) than as Class 2.

### Complications, all-cause mortality and healthcare cost

In the whole cohort (*n*=2886), the most frequent complications were CVD (20.6%), severe hypoglycaemia (13.0%), cancer (11.8%) and ESKD (6.0%), and 16.3% died during the follow-up period (Table 2). The distribution of the frequency of CVD prior to CKD onset was similar among all four classes (*p*=0.506). Class 1, characterised by YOD, had the highest frequency of severe hypoglycaemia (20.2%) and ESKD (11.5%). Class 4 (middle-aged onset) had the highest frequency of CVD (23.6%), with most of the events (15.1%) occurring after CKD onset.

**Table 2:** Diabetes-related complications, all-cause mortality and healthcare costs during follow-up, by latent class

Variable	Overall	Class 1	Class 2	Class 3	Class 4	*p* value
Diabetes-related complications and all-cause mortality, *n* (%)						
*N*	2886	529 (18.3)	612 (21.2)	979 (33.9)	766 (26.5)	
Severe hypoglycaemia	374 (13.0)	107 (20.2)	42 (6.9)	125 (12.8)	100 (13.1)	<0.001
CVD	594 (20.6)	121 (22.9)	122 (19.9)	170 (17.4)	181 (23.6)	0.006
CVD prior to CKD onset	243 (8.4)	51 (9.6)	54 (8.8)	73 (7.5)	65 (8.5)	0.506
CVD after CKD onset	351 (12.2)	70 (13.2)	68 (11.1)	97 (9.9)	116 (15.1)	0.007
Cancer	341 (11.8)	60 (11.3)	65 (10.6)	127 (13.0)	89 (11.6)	0.525
ESKD	172 (6.0)	61 (11.5)	5 (0.8)	60 (6.1)	46 (6.0)	<0.001
Death	470 (16.3)	99 (18.7)	105 (17.2)	155 (15.8)	111 (14.5)	0.205
PPPY healthcare costs, US$, mean (SD)						
*N*	24,819	4269	5193	8903	6454	
Total healthcare costs	4395 (11,947)	6087 (15,519)	3822 (9816)	4260 (11,725)	3923 (10,957)	<0.001
Cost before CKD onset	834 (5898)	956 (7077)	810 (5388)	970 (6840)	587 (3218)	<0.001
Costs of CKD onset and after onset	3561 (14,498)	5131 (18,317)	3012 (11,767)	3291 (14,314)	3336 (13,494)	<0.001
OPD costs	1086 (1492)	1396 (1789)	933 (1175)	1077 (1588)	1017 (1332)	<0.001
Acute care costs	2157 (6790)	3025 (8586)	1894 (5839)	2123 (7210)	1842 (5362)	<0.001
HDU costs	83 (1576)	127 (1413)	59 (709)	54 (1000)	112 (2538)	0.022
ICU costs	163 (2734)	289 (3648)	90 (1558)	117 (1996)	202 (3532)	0.001
Rehabilitation and infirmary costs	789 (5399)	1126 (8129)	775 (4318)	728 (5085)	661 (4197)	<0.001
Psychiatric costs	47 (1301)	38 (886)	8 (241)	92 (1993)	22 (674)	<0.001
A&E costs	58 (154)	73 (171)	56 (148)	52 (150)	56 (150)	<0.001

A&E, accident and emergency; CKD, chronic kidney disease; CVD, cardiovascular disease; ESKD, end-stage kidney disease; HDU, high-dependency unit; ICU, intensive care unit; OPD, outpatient department; SD, standard deviation

Among the 24,819 cost records, the mean (SD) total PPPY healthcare costs were US$4395 (US$11,947), with acute care costs being the highest (US$2157 [US$6790]), followed by OPD costs (US$1086 [US$1492]) (Table 2). Class 1 had the highest total PPPY costs of US$6087 (US$15,519), followed by Class 3 (US$4260 [US$11,725]), Class 4 (US$3923 [US$10,957]) and then Class 2 (US$3822 [US$9816]). Class 3 incurred the highest psychiatric care costs (US$92 [US$1993]). Before CKD onset, the total costs for the four classes ranged from US$587 (US$3218) in Class 4 to US$970 (US$6840) in Class 3. The cost increased after CKD onset, ranging from US$3012 (US$11,767) in Class 2 to US$5131 (US$18,317) in Class 1.

### PPPY healthcare cost trajectories

**Fig. 1:**
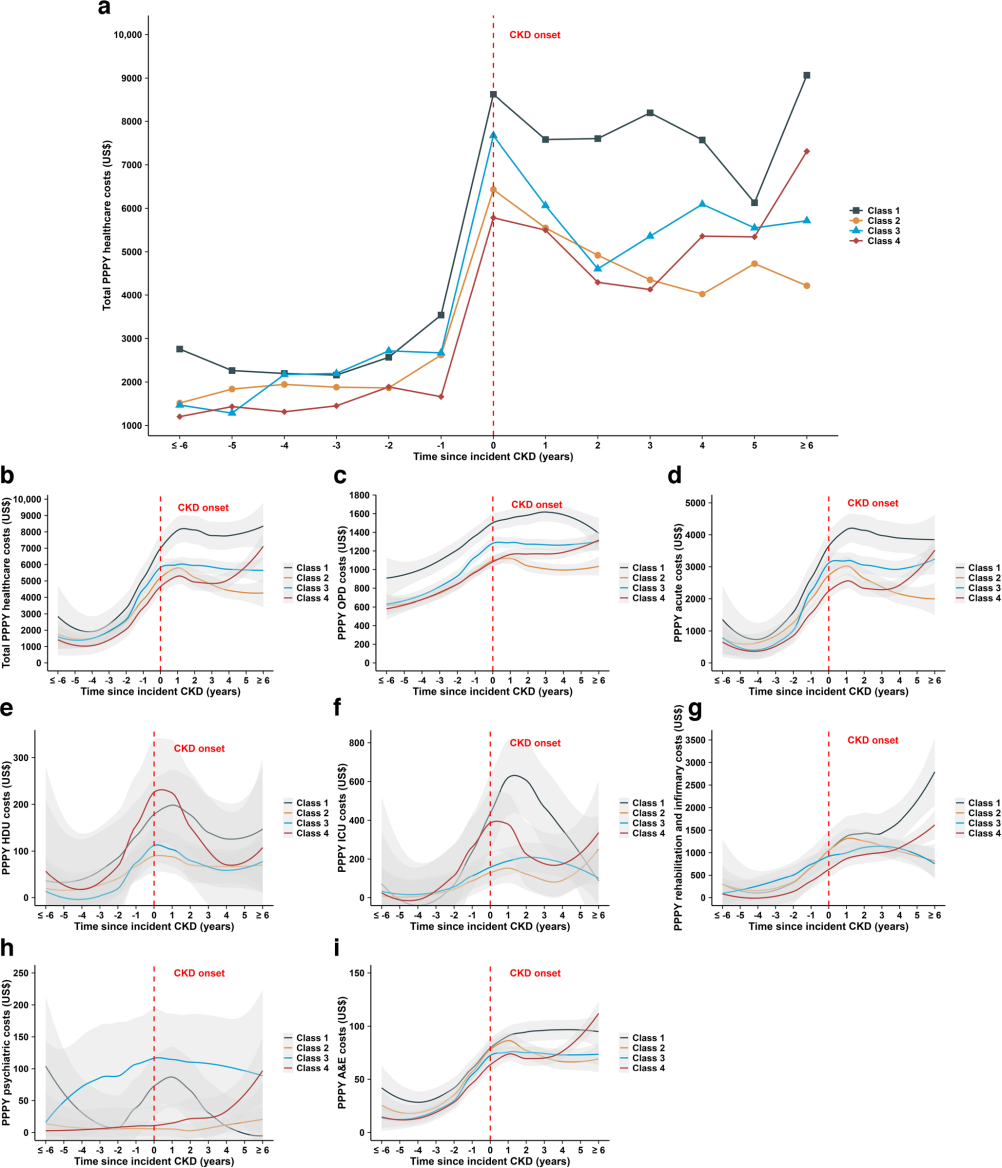
The trajectories of mean annual healthcare costs before and after incident CKD, by latent class. The trajectories of (**a**) total PPPY healthcare costs and (**b**–**i**) PPPY cost components before and after incident CKD, by latent class: (**b**) total PPPY healthcare costs; (**c**) OPD care costs; (**d**) acute care costs; (**e**) HDU care costs; (**f**) ICU care costs; (**g**) rehabilitation and infirmary care costs; (**h**) psychiatric care costs; and (**i**) A&E care costs. A solid line indicates the LOESS fit to the smoothed trajectories across the four latent classes. Shaded areas represent 95% confidence bands for the LOESS curves. If cost data contain zero values or exhibit substantial skewness, the LOESS CIs may be unstable near the boundary, which does not imply negative costs. A&E, accident and emergency; CI, confidence interval; CKD, chronic kidney disease; HDU, high-dependency unit; ICU, intensive care unit; LOESS, locally estimated scatterplot smoothing; OPD, outpatient department; PPPY, per-patient-per-year

The mean trajectories of total PPPY healthcare costs across the four classes before and after CKD onset are shown in Fig. 1a. In the period prior to CKD onset (≤–6 to −1 years), there were no significant cost differences across classes. After CKD onset, there were significant cost differences in overall healthcare costs and across the majority of healthcare service subtypes. At CKD onset, Class 1 had the highest costs (US$8625 [US$17,527]), followed by Class 3 (US$7673 [US$16,490]), Class 2 (US$6431 [US$13,511]) and then Class 4 (US$5783 [US$16,035]). After CKD onset, each class follows a distinct trend. The total PPPY costs remained the highest in Class 1, whereas Class 2 showed gradual decline. The rising overall healthcare costs in the year of CKD onset reflect hospital costs related to CKD and to the burden of existing comorbidities. Smoothed trajectories of PPPY cost components are depicted in Fig. 1b–i. Class 1 showed a general increase in costs during the year of CKD onset across all components; Class 2 exhibited a pronounced rise in acute, rehabilitation and infirmary care, and A&E costs; Class 3 displayed distinct increases in OPD, acute and psychiatric care costs; and Class 4 experienced a notable increase in HDU and ICU care costs.

### Hierarchical generalised linear mixed models of latent classes with healthcare costs

ZI-GLMM results (Table 3) indicated distinct healthcare cost utilisation patterns across four classes. The reported exp(b) values (adjusted cost ratio [95% CI]) reflect relative cost differences versus Class 2. Values >1 denote higher costs than Class 2. Class 1 exhibited significant adjusted increases in total costs (43%, *p*<0.001), including OPD (41%, *p*<0.001), acute care (25%, *p*<0.001), ICU (54%, *p*<0.05), psychiatry care (230%,* p*<0.05) and A&E (8%, *p*<0.05) costs relative to Class 2. Class 3 showed significant elevations in total, OPD and psychiatric care costs, while Class 4 demonstrated higher ICU and psychiatric care costs (ESM Table 11). Sensitivity analyses using winsorised costs and HLM yielded attenuated but generally robust estimates (ESM Tables 12–14). HLM provided more intuitive absolute cost estimates: Class 1 had the highest total estimates (standard error) of PPPY costs at US$1723 (US$383, *p*<0.001), followed by Class 3 (US$738 [US$337], *p*<0.05).

**Table 3:** Longitudinal estimates of mean per-patient-per-year (PPPY) healthcare costs (US$) using hierarchical generalised linear mixed models

Adjusted cost ratio (95% CI)	Class 2	Class 1	Class 3	Class 4
Total healthcare costs	Reference	1.43 (1.26–1.62)***	1.17 (1.04–1.30)**	1.00 (0.89–1.11)
OPD care costs	Reference	1.41 (1.31–1.52)***	1.16 (1.09–1.24)***	1.07 (1.00–1.14)
Acute care costs	Reference	1.25 (1.11–1.42)***	1.06 (0.95–1.18)	0.96 (0.86–1.07)
HDU care costs	Reference	1.18 (0.83–1.68)	0.90 (0.64–1.26)	1.15 (0.83–1.60)
ICU care costs	Reference	1.54 (1.04–2.29)*	1.37 (0.94–1.99)	1.55 (1.08–2.23)*
Rehabilitation and infirmary care costs	Reference	1.12 (0.93–1.34)	1.02 (0.87–1.21)	0.99 (0.84–1.17)
Psychiatric care costs	Reference	3.30 (1.28–8.47)*	7.41 (3.37–16.27)***	4.72 (1.83–12.23)**
A&E care costs	Reference	1.08 (1.01–1.15)*	1.06 (0.99–1.12)	1.00 (0.94–1.06)

Models were adjusted for alcohol use, family history of diabetes, diastolic BP, plasma albumin, alkaline phosphatase, eGFR, prior severe hypoglycaemia, prior CVD, prior cancer and EQ-5D-3L (mobility, self-care, usual activities, pain/discomfort and anxiety/depression). Psychiatric care cost exhibits high zero-inflation. The model was simplified by including a year-varying random slope only at the individual level, while adjusting for the variables mentioned earlier, except for alcohol use, plasma albumin and alkaline phosphatase

***p<0.001, **p<0.01, *p<0.05

A&E, accident and emergency; CI, confidence interval; CVD, cardiovascular disease; eGFR, estimated glomerular filtration rate; EQ-5D-3L, EuroQol five-dimensional three-level questionnaire; HDU, high-dependency unit; ICU, intensive care unit; OPD, outpatient department

## Discussion

### Main findings

In this 12-year prospective analysis of Chinese individuals with type 2 diabetes and incident CKD, we identified four distinct classes based on demographic and clinical profiles using LCA. These classes had different baseline characteristics and PPPY healthcare cost trajectories during follow-up. Among the four classes, total PPPY costs were the highest in YOD (Class 1) and lowest in old-age-onset type 2 diabetes (Class 2). Comorbid psychiatric conditions and poor HRQoL also increased the healthcare burden. Although Class 3 (middle-aged onset) had fewer baseline comorbidities and medications, the long-term healthcare cost in this class exceeded that of their middle-aged peers who had baseline morbidities and more treatment at baseline (Class 4). This paradox suggested that less intensified early treatment might lead to higher long-term costs with subsequent occurrence of comorbidities. There was a substantially higher clinic-based and hospitalisation cost burden in those with pre‑existing CKD than in those with incident CKD. These findings underscore the progressive clinical and economic burden of CKD in type 2 diabetes as individuals transition from a stage of newly-diagnosed to one with multiple comorbidities.

Before CKD onset, total PPPY healthcare costs were low and largely indistinguishable across the four classes. This pre‑onset similarity is consistent with the natural, silent and progressive nature of individuals with diabetes who are often managed in general medical clinics without CKD‑specific care protocol or resources [1]. Costs began rising approximately 3–5 years before CKD onset, most likely reflecting emerging renal‑related comorbidities, such as microalbuminuria [25], and peaked at the diagnosis of CKD due to intensive investigations and management. Following CKD onset, cost trajectories separated across classes, based on their distinct baseline clinical and treatment profiles. Despite their similar healthcare cost profiles at baseline, this post‑CKD-onset divergence highlights the benefit of using clusters of multidimensional data to identify participant subgroups to inform targeted, risk‑based strategies for mitigating long‑term costs.

### Association of YOD with the highest healthcare costs

While ageing is a significant factor in the growing prevalence of diabetes, the increasing YOD burden is particularly concerning [26]. Prolonged exposure to abnormal milieu in YOD increased lifetime risks of cardiorenal disease and premature mortality with personal and societal impacts [26]. A 30-year follow-up of the UK Prospective Diabetes Study found that young age at diagnosis was linked to an increased risk of all diabetes-related complications, especially in individuals with persistently unstable glycaemic management [27]. The clinical features of Class 1 members, characterised by mostly non-obese YOD with suboptimal glycaemic management, many of whom were treated with insulin at baseline, strongly suggests that beta cell dysfunction might be a key driver in their disease trajectory [28]. Until recently, islet autoantibody and C‑peptide measurements were not routine measurements in public systems to classify pathophysiological profiles.

Despite increasing epidemiological reports on YOD [26], none investigated the pattern of healthcare utilisation and costs in this high risk population. Our study found that the high costs in Class 1 during CKD onset were driven by broad cost increases across outpatient, acute, ICUs, psychiatry and A&E care. This analysis confirms the vulnerability of those with YOD to long-term, high healthcare costs. In our ongoing PRISM trial [29], we have reported complex phenotypes in individuals with YOD, who had a myriad of familial, genetic and life-course factors accompanied by a high frequency of depression. This heterogeneity calls for a collaborative care model between endocrinologists and primary-care physicians, with regular assessments followed by intensive and holistic care. Using the HKDR, we modelled that treating multiple risk factors to low targets in YOD might reduce the lifetime hospitalisation risk by 50% [30]. Taken together, these data call for the early detection of individuals at risk of YOD for prevention and intensive treatment to reduce long-term costs.

### Association of old-age-onset type 2 diabetes with the lowest healthcare costs

The global incidence and prevalence of type 2 diabetes are increasing in all age groups, with the oldest age group having the highest prevalence, accompanied by functional decline and institutionalisation before their eventual demise [31]. In this study, Class 2 was characterised by old-age-onset type 2 diabetes with the shortest diabetes duration, moderate comorbidities, better glycaemic management and multiple medications. Counter-intuitively, Class 2 had the lowest total costs among the four classes, with the lowest frequency of severe hypoglycaemia and ESKD events, which were key drivers of diabetes-related costs [7]. In these older individuals, 60% of their total healthcare costs were attributed to inpatient care, of which 93% was due to acute short-stay hospitalisations [32], with elevated HbA_1c_ as a major risk factor for hospitalisation [33]. In Hong Kong, the territory-wide diabetes risk assessment programme, which emphasises education by nurses, might benefit these older individuals most, who tend to be more adherent than younger groups [34].

### Association of early intensified interventions with reduced future costs

Observational and intervention studies have confirmed that early intensified intervention in type 2 diabetes has legacy effects in reducing long-term complications and improving socioeconomic outcomes [35, 36]. Among the four classes, Class 3 (middle-aged onset) was characterised by unstable glycaemic management, fewer comorbidities and lower use of medications, including renin–angiotensin system inhibitors (RASis), statins and aspirin. Although they appeared to be ‘low risk’ at baseline, Class 3 members incurred higher healthcare costs after CKD onset than those in Class 4, who had more comorbidities and higher baseline use of medications. This likely reflects delayed treatment intensification in at-risk individuals with less evident clinical features, albeit with legacy adverse effects on long-term renal outcome and increased healthcare costs. Early initiation of RASi and statin therapy in individuals with type 2 diabetes may reduce renal interstitial fibrosis and the risk of incident CKD [37, 38]. In the absence of distinguishable clinical features, genetic testing may identify such individuals prior to the onset of clinical features, enabling timely treatment to mitigate downstream complications [39, 40].

These findings echo the European Association for the Study of Diabetes/American Diabetes Association 2022 guidelines, which recommend stricter glycaemic targets (<7.0–7.5% [53–58 mmol/mol]) for individuals with few comorbidities [41]. In an HKDR propensity-score matching analysis, we reported that intensifying treatment within 2 years of diagnosis and before HbA_1c_ reached >7.5% (58 mmol/mol) reduced glycaemic variability and delayed insulin initiation with marked (24%–62%) risk reduction in severe hypoglycaemia, cardiovascular-renal events and mortality [42, 43]. Our results reinforce that early intensified treatment with strict glycaemic management is crucial in reducing downstream complications and mitigating future healthcare costs.

### Association of comorbid mental illness and type 2 diabetes with high healthcare costs

Our group was amongst the first to report the high depression burden in individuals with type 2 diabetes and its independent association with CVD and all-cause death, adjusted for HRQoL [44]. The co-occurrence of depression and diabetes incurred 4.5-fold higher total healthcare costs than diabetes alone [45]. In Classes 1, 3 and 4, costs due to psychiatric care were three to seven times higher than in Class 2. Winsorised data confirmed that the middle-aged onset classes (Classes 3 and 4) still had three times higher costs after adjustment for covariables. Elevated costs may stem from prior medical history, poor HRQoL and suboptimal adherence to self-care practices with unstable glycaemic management [45, 46]. Overall, our results call for screening for depression, especially in young and middle-aged individuals and those with suboptimal risk-factor control for holistic management and cost reduction.

### Association of poor HRQoL and increased healthcare costs

The structured HKDR assessment helped identify attributes associated with healthcare cost trajectories. Consistent with other reports [47], we also found correlations between healthcare costs and poor HRQoL, mainly related to difficulties in daily physical activities often limited by complications. In a randomised controlled trial, intensive lifestyle intervention with increased physical activity and balanced nutrition reduced body weight, BP, blood lipids and glucose [48] and reduced the risk of incident CKD stage 3 (<60 ml/min per 1.73 m^2^) [49] and depression [23]. Despite advances in the development of pharmaceutical care for CKD prevention and treatment [10, 23], our data suggest that self-care and mental wellness equally influence long-term costs in support of holistic diabetes management.

### Strengths and limitations

This is the first study applying LCA to an ongoing register with minimal missing data. This data granularity, including psychosocial-behavioural factors and HRQoL, allowed us to dissect the heterogeneity of different components of costs associated with CKD. Limitations included the fact that our cohort consisted of individuals who were referred to a diabetes centre for quality improvement assessment and therefore potentially had a higher risk than primary-care patients. Second, structured assessments, including the collection of multidimensional data, were undertaken every 2–3 years, primarily for quality improvement, which limited the analysis of latent-class changes over time. Third, we included only publicly-funded care, although about 90% of individuals with type 2 diabetes receive public services due to subsidised costs [50]. Fourth, assigning individuals to latent classes by maximum probability in LCA constitutes an artificial categorisation that excludes their probabilities of belonging to other latent classes. This may introduce classification bias and attenuate associations with other variables [17].

In conclusion, we identified four distinct classes of individuals with type 2 diabetes and incident CKD, with the highest costs incurred by individuals diagnosed with type 2 diabetes at a young age or in middle age. For those with middle-aged onset, few baseline morbidities and low medication use, high long-term costs in the post-onset period emphasised the progressive nature of diabetes. This paradox suggested that regular assessments might identify gaps for early intensified treatment and long-term organ protection. In individuals with old-age onset, despite having comorbidities, their shorter disease duration and intensified treatment at baseline were associated with better outcomes than the young-age group. The highest costs in YOD call for urgent targeted programmes to prevent and intensively manage YOD. The role of mental illness and self-care practice reinforces the importance of psychological-behavioural and pharmaceutical care in changing the cost trajectories in type 2 diabetes with CKD.

## Supplementary Information

Below is the link to the electronic supplementary material.

ESM (PDF 885 KB)

## Data Availability

The participant-level data used and/or analysed during the current study are not available, since consent was not been obtained for data sharing. Summary data may be available from the corresponding author on reasonable request.

## Abbreviations

A&E: Accident and emergency
ACEi: Angiotensin-converting enzyme inhibitor
aOR: Adjusted odds ratio
ARB: Angiotensin II receptor blockers
BIC: Bayesian information criterion
BP: Blood pressure
CI: Confidence interval
CKD: Chronic kidney disease
CVD: Cardiovascular disease
ECI: Elixhauser Comorbidity Index
eGFR: Estimated glomerular filtration rate
EMR: Electronic medical record
EQ-5D-3L: EuroQol five-dimensional three-level questionnaire
ESKD: End-stage kidney disease
HA: Hospital Authority
HbA_1c_: Haemoglobin A_1c_
HDU: High-dependency unit
HKDR: Hong Kong Diabetes Register
HLM: Hierarchical linear modelling
HRQoL: Health-related quality of life
ICU: Intensive care unit
IQR: Interquartile range
LCA: Latent class analysis
LOESS: Locally estimated scatterplot smoothing
MNL: Multinomial logistic regression
OPD: Outpatient department
PPPY: Per-patient-per-year
RASi: Renin–angiotensin system inhibitor
SD: Standard deviation
UACR: Urinary albumin/creatinine ratio
YOD: Young-onset type 2 diabetes
ZI-GLMM: Zero-inflated generalised linear mixed model

## Authors’ relationships and activities

APSK has received research grants and/or speaker honoraria from Abbott, Astra Zeneca, Bayer, Boehringer Ingelheim, Eli Lilly, Kyowa Kirin, Merck Serono, Nestle, Novo Nordisk, Pfizer and Sanofi. AOYL reported receiving grants and/or honoraria for consultancy or giving lectures from Amgen, AstraZeneca, Boehringer Ingelheim, Merck Sharp & Dohme, Novartis, Novo Nordisk and Sanofi. RCWM reported receiving grants and/or honoraria for consultancy or giving lectures from AstraZeneca, Bayer, Boehringer Ingelheim, Eli Lilly, Pfizer and Takeda and is a member of the editorial board of *Diabetologia*. L-LL reported receiving grants and/or honoraria for consultancy or giving lectures from AstraZeneca, Abbott, Boehringer Ingelheim, GSK, Novartis, Novo Nordisk, Viatris and Zuellig Pharma. JCNC has received research grants through her affiliated institutions and/or honoraria for consultancy or giving lectures from Applied Therapeutics, AstraZeneca, Bayer, Boehringer Ingelheim, Celltrion, Eli Lilly, Hua Medicine, Merck, Merck Sharp & Dohme, Novo Nordisk, Novartis, Pfizer, Servier, Sanofi and Viatris. JNML has received a research grant from Roche Diagnostics Hong Kong Limited for a project unrelated to this manuscript. All other authors declare that there are no relationships or activities that might bias, or be perceived to bias, their contribution to this manuscript.
