# Supplementary material for: Differential healthcare costs in individuals with type 2 diabetes and incident chronic kidney disease in Hong Kong: a latent class trajectory analysis
Source: Diabetologia. 2026 Mar 18;69(7):1792–805. doi: 10.1007/s00125-026-06698-2 (PMC13236821; doi:10.1007/s00125-026-06698-2)
Supplement: Supplementary file 1 — ESM (PDF 885 KB) [file 125_2026_6698_MOESM1_ESM.pdf]

Differential healthcare costs in individuals with type 2 diabetes and incident chronic kidney disease in Hong Kong: a latent class trajectory analysis

Electronic Supplementary Material

ESM Table 1. ICD-9 admissions and procedure codes for categorization of diabetes complication 2
ESM Table 2. Definitions of covariates 4
ESM Table 3. Unit healthcare costs categorised by inpatient and outpatient service types listed in the Hong Kong Gazette and Hospital Authority Ordinance published in 2023........ 8
ESM Table 4. List of indicator variables used in analyses 9
ESM Table 5. Missing data distribution of baseline characteristics before imputation........10
ESM Table 6. Baseline characteristics of individuals with T2D during structured assessment enrolled in the HKDR in 2007–2019 12
ESM Table 7. Diabetes-related complications, all-cause mortality and healthcare costs of individuals with T2D with pre-existing or incident CKD 18
ESM Table 8. Fit statistics for latent class models from two to six classes 19
ESM Table 9. Definitions and derivation of terminologies used for naming of latent classes 20
ESM Table 10. Covariates associated with class membership identified using multinomial logistic regression relative to Class 2 21
ESM Table 11. Longitudinal estimates of mean per-patient-per-year (PPPY) healthcare costs (US$) using hierarchical generalised linear mixed models 24
ESM Table 12. Sensitivity analyses of longitudinal estimates of mean per-patient-per-year (PPPY) healthcare costs (US$) using hierarchical generalised linear mixed models based on winsorised costs........26
ESM Table 13. Sensitivity analyses of longitudinal estimates of mean per-patient-per-year (PPPY) healthcare costs (US$) using hierarchical linear models based on original costs 28
ESM Table 14. Sensitivity analyses of longitudinal estimates of mean per-patient-per-year (PPPY) healthcare costs (US$) using hierarchical linear models based on winsorised costs 30
ESM Fig. 1. Plots of mean and standard deviation of the imputed variables against iteration number for the imputed data 32
ESM Fig. 2. Flow chart of the study........33
ESM Fig. 3. Distribution of latent class indicators based on class assignment in the four-class model........34

ESM Table 1. ICD-9 admissions and procedure codes for categorization of diabetes complication

**Severe hypoglycaemia**	1) Diabetes with other coma (250.3)<br>2) Diabetes with other specified manifestations (250.8)<br>3) Hypoglycaemia, unspecified (251.2)
**Cardiovascular disease**	1) Coronary heart disease<br>• Acute myocardial infarction (410)<br>• Other acute and subacute forms of ischemic heart disease (411)<br>• Old myocardial infarction (412)<br>• Angina pectoris (413)<br>• Other forms of chronic ischemic heart disease (414)<br>• Coronary revascularization (procedure code: 36)<br>• Percutaneous transluminal coronary angioplasty or coronary atherectomy (procedure code: 00.66)<br>2) Stroke<br>• Subarachnoid hemorrhage (430)<br>• Intracerebral hemorrhage (431)<br>• Other and unspecified intracranial hemorrhage (432)<br>• Occlusion and stenosis of precerebral arteries (433)<br>• Occlusion of cerebral arteries (434)<br>• Acute, but ill-defined, cerebrovascular disease (436)<br>• Other and ill-defined cerebrovascular disease (437)<br>• Late effects of cerebrovascular disease (438)<br>3) Peripheral vascular disease (excluding amputation of lower limb)<br>• Diabetes with peripheral circulatory disorders (250.7)<br>• Gangrene (785.4)<br>• Peripheral angiopathy in diseases classified elsewhere (443.81)<br>• Peripheral vascular disease, unspecified (443.9)<br>• Procedures: Other (peripheral) vascular shunt or bypass (procedure code 39.29); insertion of non-drug-eluting peripheral vessel stent(s) (39.90); 38.08, 38.18, 38.38, 38.48, 38.68, 38.88, 39.25, 39.49, 39.56, 39.57, 39.58, 39.59, 39.99; 00.55, 17.56, 39.50, 39.79
**Diabetic eye disease**	1) Diabetes with ophthalmic manifestations (250.5)<br>2) Diabetic retinopathy (362.0)<br>3) Vitreous hemorrhage (379.23)
**Peripheral neuropathy**	1) Diabetes with neurological manifestations (250.6)<br>2) Polyneuropathy in diabetes (357.2)
**Cancer**	Neoplasms (140-208)
**Chronic kidney disease**	1) End-stage renal disease<br>2) eGFR<60 ml/min/1.73m^2, exclude acute kidney failure<br>• for new event, 2 eGFR<60 ml/min/1.73m^2 separated by 90-365 days

	• for history, the eGFR at baseline or the latest eGFR before baseline <60 ml/min/1.73m^2
**End-stage kidney disease**	1) eGFR<15 ml/min/1.73m^2, exclude acute kidney failure<br>• for new event, 2 eGFR<15 ml/min/1.73m^2 separated by 90-365 days<br>• for history, the eGFR at baseline or the latest eGFR before baseline <15 ml/min/1.73m^2<br>2) Haemodialysis dialysis (procedure code 39.95) with diagnosis of chronic kidney disease (585) or Renal failure (586)<br>3) Peritoneal dialysis (procedure code 54.98)<br>4) Transplant of kidney (procedure code 55.6)<br>5) Complications of transplanted kidney (996.81)<br>6) Persons with a condition influencing their health status; organ or tissue replaced by transplant; kidney (V42.0)

eGFR, Estimated glomerular filtration rate.

ESM Table 2. Definitions of covariates

Variables	Categories (if categorical)	Remarks
**Personal characteristics**		
Follow-up (years)		
Sex	Female<br>Male	
Age at assessment (years)	< 60<br>≥ 60	
Age at diagnosis (years)	< 40<br>40-60<br>≥ 60	
Duration of diabetes (years)	< 5<br>5- 10<br>10- 15<br>≥ 15	
Education level	Primary, illiterate or others<br>Middle or high school<br>College or above	
Occupation status	Employed (full-time/part-time)<br>Housewife/students/unemployed<br>Retired	
Family history of diabetes	False<br>True	
**Lifestyle factors**		
Consumption of alcohol	Never<br>Ex-drinker<br>Current drinker	
Smoking	Never<br>Ex-smoker<br>Current smoker	
Frequency of physical activity (times/week)	No regular activity<br>Less than three times/week<br>Three or four times/week<br>Five times/week<br>More than five times/week	Vigorous exercise corresponding to brisk walking of > 30 minutes.
Adherence to balanced diet in last 3 months	No<br>Occasional<br>Yes	
Self-monitoring of blood glucose	False<br>True	The result report indicates the selection of True.
**Clinical characteristics**		
Central obesity (waist circumference ≥90 cm in men and ≥85 cm in women)	False<br>True	The result report indicates the selection of True.
Body mass index (BMI) (kg/m^2)	< 25<br>25-30<br>≥ 30	
Haemoglobin A_{1c}	< 7% (< 53 mmol/mol)<br>≥ 7% (≥ 53 mmol/mol)	

Variables	Categories (if categorical)	Remarks
Fasting plasma glucose (mmol/l)	< 7<br>≥ 7	
Diastolic blood pressure (mmHg)	< 75<br>75-85<br>≥ 85	
Systolic blood pressure (mmHg)	< 125<br>125-140<br>≥ 140	
Total cholesterol (mmol/l)	< 5.2<br>≥ 5.2	
Triglycerides (mmol/l)	< 1.69<br>1.69-2.26<br>≥ 2.26	
High-density lipoprotein (HDL) cholesterol (mmol/l)	< 1.3<br>1.3-1.55<br>≥ 1.55	
Low-density lipoprotein (LDL) cholesterol (mmol/l)	< 1.4<br>1.4-1.8<br>1.8-2.6<br>≥ 2.6	
Haemoglobin (g/l)	Normal<br>Anaemia	Anaemia was defined by World Health Organization (WHO) criteria: Haemoglobin < 120 g/l for women and <130 g/l for men.
Plasma albumin (g/l)	< 35<br>35-50<br>≥ 50	
Alanine aminotransferase (mmol/l)	< 10<br>10-40<br>≥ 40	
Alkaline phosphatase (mmol/l)	< 44<br>44-147<br>≥ 147	
Bilirubin (µmol/l)	< 5<br>5-21<br>≥ 21	
Estimated glomerular filtration rate (eGFR, ml/min/1.73m^2)	60-90<br>≥ 90	Calculated using the Chronic Kidney Disease Epidemiology Collaboration equation.<br>People with a history of CKD were excluded from this study (eGFR < 60 ml/min/1.73m^2).
Urinary albumin/creatinine ratio (UACR, mg/mmol)	< 3<br>3-30<br>≥ 30	

Variables	Categories (if categorical)	Remarks
**Medical treatments**		
Glucose-lowering drugs	False<br>True	The result report indicates the selection of True. Alpha-glucosidase inhibitor (AGIs), dipeptidyl peptidase 4 (DPP-4) inhibitor, meglitinide, metformin, sodium-glucose cotransporter-2 (SGLT2) inhibitor, sulphonylurea, thiazolidinedione.
SGLT2 inhibitor	False<br>True	The result report indicates the selection of True.
GLP-1 RAs (glucagon-like peptide-1 receptor agonists)	False<br>True	The result report indicates the selection of True.
Insulin	False<br>True	The result report indicates the selection of True.
Blood pressure lowering drugs (excluding renin angiotensin system inhibitors, RASi)	False<br>True	The result report indicates the selection of True. Alpha blocker, beta blocker, calcium channel blocker, hydralazine, methyldopa, nitrate, thiazide.
RASi (including angiotensin converting enzyme inhibitors, ACEi and angiotensin receptor blockers, ARB)	False<br>True	The result report indicates the selection of True.
ACEi	False<br>True	The result report indicates the selection of True.
ARB	False<br>True	The result report indicates the selection of True.
Lipid-lowering drugs (excluding statins)	False<br>True	The result report indicates the selection of True. Ezetimibe, cholestyramine, fibrate.
Statins	False<br>True	The result report indicates the selection of True.
Aspirin	False<br>True	The result report indicates the selection of True.
**Medical history**		
Sensory neuropathy	False<br>True	The result report indicates the selection of True. Two of three abnormal features: symptoms (subjective) in the feet, and signs (objective) by reduced vibration sensation

Variables	Categories (if categorical)	Remarks
		to tuning fork or reduced pressure sensation to monofilament.
Diabetic retinopathy	False<br>True	The result report indicates the selection of True. Retinal photography was used to define diabetic retinopathy read by endocrinologists or trained fellows. Severe retinopathy was defined as proliferative retinopathy, maculopathy or advanced eye disease.
Prior severe hypoglycaemia (requiring assistance by third person or medical care)	False<br>True	The result report indicates the selection of True.
Prior cardiovascular disease	False<br>True	The result report indicates the selection of True.
Prior cancer	False<br>True	The result report indicates the selection of True.
Elixhauser Comorbidity Index,<br>ECI score (range: <0–30)	No comorbidity (0)<br>Moderate ECI (<0 or 1-5)<br>High ECI (> 5)	The van Walraven (VW) weighting algorithm was employed to calculate the ECI. A VW-weighted score of 0 indicated no comorbidity, scores less than 0 or between 0 and 5 were classified as moderate ECI, while scores of 5 or greater were considered indicative of high ECI.
**EQ-5D-3L**		
Mobility	No problems<br>Some or severe problems	
Self-care	No problems<br>Some or severe problems	
Usual activities	No problems<br>Some or severe problems	
Pain/discomfort	No problems<br>Some or severe problems	
Anxiety/depression	No problems<br>Some or severe problems	

EQ-5D-3L, EuroQol five-dimensional three-level questionnaire.

ESM Table 3. Unit healthcare costs categorised by inpatient and outpatient service types listed in the Hong Kong Gazette and Hospital Authority Ordinance published in 2023

**Hospital service type**	**Unit hospital cost (US$)**
**Inpatient**	**Per day**
Acute, convalescent, rehabilitation or infirmary	654
High dependency ward/unit	1750
Intensive care ward/unit	3128
Psychiatric hospital	300
**Outpatient**	**Per attendance/visit**
Accident & Emergency	158
Community allied health service	222
Community nursing service (general)	69
Community nursing service (psychiatry)	199
Day procedure and treatment at Clinical Oncology Clinic	115
Day procedure and treatment at Ophthalmic Clinic	93
Day procedure and treatment in ambulatory facility	654
General clinic	57
Specialist outpatient (including allied health service clinic)	153
Geriatric day hospital	251
Psychiatric day hospital	162
Rehabilitation day hospital	169
Injection or dressing at clinic/hospital	13

Average inpatient maintenance cost per day includes charges of all necessary clinical, biochemical, and pathology investigations, such as consultations, diagnostic imaging, and other examinations, administration of any required vaccines, general nursing care and prescriptions during the stay. The unit costs for inpatient and outpatient healthcare services were published in the Hong Kong Gazette and Hospital Authority Ordinance in 2023, the year when our analysis was carried out, thus inflation adjustments was not necessary.

ESM Table 4. List of indicator variables used in analyses

Indicator variables	Categories
Age at diagnosis (years)	< 40<br>40-60<br>≥ 60
Duration of diabetes (years)	< 5<br>5- 10<br>10- 15<br>≥ 15
Frequency of physical activity (times/week)	No regular activity<br>Less than three times/week<br>Three or four times/week<br>Five times/week<br>More than five times/week
Adherence to balanced diet in last 3 months	No<br>Occasional<br>Yes
Self-monitoring of blood glucose	False<br>True
ECI score (range: <0–30)	No comorbidity (0)<br>Moderate ECI (<0 or 1-5)<br>High ECI (> 5)
Haemoglobin A_{1c}	< 7% (< 53 mmol/mol)<br>≥ 7% (≥ 53 mmol/mol)
Urine Albumin:Creatinine Ratio (mg/mmol)	< 3<br>3-30<br>≥ 30
Glucose-lowering drugs	False<br>True
Insulin	False<br>True
Blood pressure lowering drugs (excluding renin angiotensin system inhibitors, RASi)	False<br>True
RASi (including ACEi and ARB)	False<br>True
Statins	False<br>True
Aspirin	False<br>True

ACEi, Angiotensin-converting enzyme inhibitors; ARB, Angiotensin II receptor blockers; ECI, Elixhauser Comorbidity Index; RASi, Renin-angiotensin system inhibitor.

ESM Table 5. Missing data distribution of baseline characteristics before imputation

	Missing rate (n [%])
N	23247
**Personal characteristics**	
Sex	0 (0)
Age at assessment	0 (0)
Age at diagnosis	126 (0.5)
Duration of diabetes	126 (0.5)
Education level	85 (0.4)
Occupation status	12 (0.1)
Family history of diabetes	1606 (6.9)
**Lifestyle factors**	
Consumption of alcohol	9 (0)
Smoking	5 (0)
Frequency of physical activity	941 (4)
Adherence to balanced diet in last 3 months	61 (0.3)
Self-monitoring of blood glucose	1647 (7.1)
**Clinical characteristics**	
Waist circumference	86 (0.4)
Body mass index	79 (0.3)
Haemoglobin A_{1c}	43 (0.2)
Fasting plasma glucose	87 (0.4)
Diastolic blood pressure	10 (0)
Systolic blood pressure	6 (0)
Total cholesterol	838 (3.6)
Triglycerides	82 (0.4)
High-density lipoprotein (HDL) cholesterol	112 (0.5)
Low-density lipoprotein (LDL) cholesterol	474 (2)
Haemoglobin	166 (0.7)
Plasma albumin	84 (0.4)
Alanine aminotransferase	112 (0.5)
Alkaline phosphatase	83 (0.4)
Bilirubin	120 (0.5)
eGFR	23 (0.1)
UACR	443 (1.9)
**Medical treatments**	
Glucose-lowering drugs	0 (0)
SGLT2i	330 (1.4)
GLP-1 RAs	330 (1.4)
Insulin	0 (0)
Blood pressure lowering drugs (excluding RASi)	0 (0)
RASi	494 (2.1)
ACEi	494 (2.1)

	Missing rate (n [%])
ARB	494 (2.1)
Lipid-lowering drugs (excluding statins)	0 (0)
Statins	1104 (4.7)
Aspirin	0 (0)
**Medical history**	
Sensory neuropathy	2 (0)
Diabetic retinopathy	16 (0.1)
Prior severe hypoglycaemia	0 (0)
Prior cardiovascular disease	0 (0)
Prior cancer	0 (0)
ECI score	0 (0)

ACEi, Angiotensin-converting enzyme inhibitors; ARB, Angiotensin II receptor blockers; eGFR, Estimated glomerular filtration rate; ECI, Elixhauser Comorbidity Index; GLP-1 RAs, Glucagon-like peptide-1 receptor agonists; RASi, Renin-angiotensin system inhibitors; SGLT2i, Sodium-glucose transporter 2 inhibitors; UACR, Urine albumin/creatinine ratio.

ESM Table 6. Baseline characteristics of individuals with T2D during structured assessment enrolled in the HKDR in 2007–2019

	Overall	Without CKD at baseline and during follow-up	Pre-existing CKD at baseline	Incident CKD during follow-up	*P* value	
					Without CKD vs. Incident CKD	Pre-existing CKD vs. Incident CKD
N	15974	10380	2708	2886		
**Personal characteristics**						
Follow-up (years) (median [IQR])	7.3 (4.6, 9.2)	8.2 (5.9, 9.5)	6.6 (4.4, 8.8)	3.6 (1.5, 5.9)	<0.001	<0.001
Sex (%)						
Female	6937 (43.4)	4559 (43.9)	1118 (41.3)	1260 (43.7)	0.802	0.073
Male	9037 (56.6)	5821 (56.1)	1590 (58.7)	1626 (56.3)		
Age at assessment (years) (mean [SD])	59.5 (11.7)	55.7 (10.6)	69.1 (10.2)	63.9 (10.0)	<0.001	<0.001
Age at assessment (years) (%)						
< 60	8355 (52.3)	6856 (66)	537 (19.8)	962 (33.3)	<0.001	<0.001
≥ 60	7619 (47.7)	3524 (34)	2171 (80.2)	1924 (66.7)		
Age at diagnosis (years) (mean [SD])	52.2 (11.5)	50.0 (10.8)	57.3 (12.0)	55.1 (11.4)	<0.001	<0.001
Age at diagnosis (years) (%)						
< 40	2105 (13.2)	1650 (15.9)	197 (7.3)	257 (8.9)	<0.001	<0.001
40-60	9821 (61.5)	6875 (66.2)	1318 (48.7)	1628 (56.4)		
≥ 60	4048 (25.3)	1855 (17.9)	1192 (44.0)	1001 (34.7)		
Duration of diabetes (years) (mean [SD])	7.3 (7.4)	5.7 (6.2)	11.6 (8.9)	8.7 (7.7)	<0.001	<0.001
Duration of diabetes (years) (%)						
< 5	7594 (47.5)	5834 (56.2)	695 (25.7)	1065 (36.9)	<0.001	<0.001
5-10	3450 (21.6)	2207 (21.3)	550 (20.3)	694 (24.0)		
10-15	2439 (15.3)	1307 (12.6)	588 (21.7)	544 (18.8)		
≥ 15	2491 (15.6)	1031 (9.9)	875 (32.3)	584 (20.2)		
Education level (%)						
Primary, illiterate or others	6374 (39.9)	3359 (32.4)	1556 (57.5)	1459 (50.5)	<0.001	<0.001
Middle or high school	7603 (47.6)	5484 (52.8)	954 (35.2)	1165 (40.4)		
College or above	1996 (12.5)	1537 (14.8)	197 (7.3)	262 (9.1)		

	Overall	Without CKD at baseline and during follow-up	Pre-existing CKD at baseline	Incident CKD during follow-up	*P* value	
					Without CKD vs. Incident CKD	Pre-existing CKD vs. Incident CKD
Occupation status (%)						
Employed (full-time/part-time)	6397 (40.0)	5256 (50.6)	424 (15.7)	717 (24.8)	<0.001	<0.001
Housewife/students/unemployed	4332 (27.1)	2595 (25.0)	823 (30.4)	914 (31.7)		
Retired	5245 (32.8)	2529 (24.4)	1461 (54.0)	1255 (43.5)		
Family history of diabetes (%)						
False	6465 (40.5)	3794 (36.5)	1382 (51.0)	1290 (44.7)	<0.001	<0.001
True	9509 (59.5)	6586 (63.5)	1326 (49.0)	1596 (55.3)		
**Lifestyle factors**						
Consumption of alcohol (%)						
Never	9005 (56.4)	5665 (54.6)	1666 (61.5)	1674 (58.0)	<0.001	<0.001
Ex-drinker	1994 (12.5)	1073 (10.3)	503 (18.6)	417 (14.5)		
Current drinker	4976 (31.1)	3642 (35.1)	538 (19.9)	795 (27.6)		
Smoking (%)						
Never	10674 (66.8)	7117 (68.6)	1676 (61.9)	1881 (65.2)	<0.001	<0.001
Ex-smoker	3309 (20.7)	1854 (17.9)	779 (28.8)	676 (23.4)		
Current smoker	1991 (12.5)	1409 (13.6)	253 (9.3)	329 (11.4)		
Frequency of physical activity (%)						
No regular activity	6422 (40.2)	4196 (40.4)	1115 (41.2)	1111 (38.5)	<0.001	0.012
Less than three times/week	2388 (14.9)	1806 (17.4)	253 (9.3)	329 (11.4)		
Three or four times/week	1387 (8.7)	938 (9.0)	208 (7.7)	241 (8.3)		
Five times/week	765 (4.8)	507 (4.9)	110 (4.1)	148 (5.1)		
More than five times/week	5013 (31.4)	2934 (28.3)	1021 (37.7)	1057 (36.6)		
Adherence to balanced diet in last 3 months (%)						
No	1724 (10.8)	1106 (10.7)	281 (10.4)	336 (11.7)	<0.001	0.005
Occasional	5833 (36.5)	3992 (38.5)	849 (31.4)	992 (34.4)		
Yes	8417 (52.7)	5281 (50.9)	1577 (58.2)	1558 (54.0)		
Self-monitoring of blood glucose (%)	10585 (66.3)	6734 (64.9)	1932 (71.3)	1919 (66.5)	0.106	<0.001
**Clinical and biochemical characteristics**						

	Overall	Without CKD at baseline and during follow-up	Pre-existing CKD at baseline	Incident CKD during follow-up	*P* value	
					Without CKD vs. Incident CKD	Pre-existing CKD vs. Incident CKD
Central obesity (%)	8862 (55.5)	5524 (53.2)	1680 (62.0)	1657 (57.4)	<0.001	<0.001
BMI (kg/m^2) (%)						
< 25	7075 (44.3)	4680 (45.1)	1091 (40.3)	1304 (45.2)	0.900	<0.001
25-30	6398 (40.0)	4103 (39.5)	1166 (43.1)	1129 (39.1)		
≥ 30	2500 (15.7)	1597 (15.4)	451 (16.6)	452 (15.7)		
Haemoglobin A_{1c} (%)						
< 7% (< 53 mmol/mol)	7266 (45.5)	5023 (48.4)	1097 (40.5)	1146 (39.7)	<0.001	0.534
≥ 7% (≥ 53 mmol/mol)	8708 (54.5)	5357 (51.6)	1611 (59.5)	1740 (60.3)		
Fasting plasma glucose (mmol/l) (%)						
< 7	6966 (43.6)	4528 (43.6)	1279 (47.2)	1159 (40.2)	<0.001	<0.001
≥ 7	9008 (56.4)	5852 (56.4)	1429 (52.8)	1727 (59.8)		
Diastolic blood pressure (mmHg) (%)						
< 75	6402 (40.1)	4150 (40.0)	1211 (44.7)	1041 (36.1)	<0.001	<0.001
75-85	5580 (34.9)	3721 (35.9)	852 (31.4)	1007 (34.9)		
≥ 85	3991 (25.0)	2509 (24.2)	645 (23.8)	837 (29.0)		
Systolic blood pressure (mmHg) (%)						
< 125	5120 (32.0)	3992 (38.5)	512 (18.9)	616 (21.4)	<0.001	<0.001
125-140	5108 (32.0)	3492 (33.6)	717 (26.5)	899 (31.2)		
≥ 140	5745 (36.0)	2896 (27.9)	1479 (54.6)	1370 (47.5)		
Total cholesterol (mmol/l) (%)						
< 5.2	11719 (73.4)	7557 (72.8)	2051 (75.8)	2111 (73.2)	0.714	0.026
≥ 5.2	4255 (26.6)	2823 (27.2)	657 (24.2)	775 (26.9)		
Triglycerides (mmol/l) (%)						
< 1.69	10447 (65.4)	7090 (68.3)	1576 (58.2)	1782 (61.7)	<0.001	0.014
1.69-2.26	2677 (16.8)	1659 (16.0)	502 (18.6)	515 (17.9)		
≥ 2.26	2850 (17.8)	1631 (15.7)	630 (23.3)	589 (20.4)		
High-density lipoprotein (HDL) cholesterol (mmol/l) (%)						

	Overall	Without CKD at baseline and during follow-up	Pre-existing CKD at baseline	Incident CKD during follow-up	*P* value	
					Without CKD vs. Incident CKD	Pre-existing CKD vs. Incident CKD
< 1.3	8095 (50.7)	5055 (48.7)	1538 (56.8)	1501 (52.0)	0.001	0.001
1.3-1.55	4603 (28.8)	3056 (29.4)	714 (26.4)	833 (28.9)		
≥ 1.55	3276 (20.5)	2269 (21.9)	455 (16.8)	552 (19.1)		
Low-density lipoprotein (LDL) cholesterol (mmol/l) (%)						
< 1.4	839 (5.2)	455 (4.4)	206 (7.6)	178 (6.2)	<0.001	0.007
1.4-1.8	1728 (10.8)	1013 (9.8)	359 (13.3)	355 (12.3)		
1.8-2.6	5716 (35.8)	3672 (35.4)	1014 (37.5)	1029 (35.6)		
≥ 2.6	7692 (48.1)	5239 (50.5)	1128 (41.7)	1324 (45.9)		
Haemoglobin (g/l) (%)						
Normal	12888 (80.7)	9243 (89)	1430 (52.8)	2215 (76.7)	<0.001	<0.001
Anaemia	3086 (19.3)	1137 (10.9)	1278 (47.2)	672 (23.3)		
Plasma albumin (g/l) (%)						
< 35	201 (1.3)	35 (0.3)	129 (4.8)	38 (1.3)	<0.001	<0.001
35-50	15415 (96.5)	10063 (97.0)	2546 (94.0)	2806 (97.2)		
≥ 50	357 (2.2)	282 (2.7)	33 (1.2)	42 (1.5)		
Alanine aminotransferase (mmol/l) (%)						
< 10	385 (2.4)	189 (1.8)	125 (4.6)	72 (2.5)	<0.001	<0.001
10-40	12765 (79.9)	8090 (77.9)	2284 (84.4)	2390 (82.8)		
≥ 40	2824 (17.7)	2101 (20.2)	299 (11.0)	424 (14.7)		
Alkaline phosphatase (mmol/l) (%)						
< 44	899 (5.6)	621 (6.0)	114 (4.2)	164 (5.7)	0.022	0.004
44-147	14884 (93.2)	9670 (93.2)	2534 (93.6)	2681 (92.9)		
≥ 147	190 (1.2)	89 (0.9)	60 (2.2)	41 (1.4)		
Bilirubin (μmol/l) (%)						
< 5	418 (2.6)	176 (1.7)	175 (6.4)	68 (2.4)	0.010	<0.001
5-21	14710 (92.1)	9598 (92.5)	2434 (89.9)	2678 (92.8)		
≥ 21	845 (5.3)	606 (5.8)	99 (3.7)	140 (4.8)		

	Overall	Without CKD at baseline and during follow-up	Pre-existing CKD at baseline	Incident CKD during follow-up	*P* value	
					Without CKD vs. Incident CKD	Pre-existing CKD vs. Incident CKD
eGFR (ml/min/1.73m^2) (%)						
< 30	493 (3.1)	0 (0)	493 (18.2)	0 (0)	NA	<0.001
30-60	2146 (13.4)	1 (0)	2145 (79.2)	0 (0)		
60-90	6336 (39.7)	3919 (37.8)	52 (1.9)	2365 (81.9)		
≥ 90	6999 (43.8)	6460 (62.2)	18 (0.7)	521 (18.1)		
UACR (mg/mmol) (%)						
< 3	9751 (61.0)	7709 (74.3)	695 (25.7)	1347 (46.7)	<0.001	<0.001
3-30	4309 (27.0)	2316 (22.3)	968 (35.7)	1025 (35.5)		
≥ 30	1915 (12.0)	356 (3.4)	1045 (38.6)	514 (17.8)		
**Medical treatments**						
Glucose-lowering drugs (%)	13590 (85.1)	8851 (85.3)	2106 (77.8)	2633 (91.2)	<0.001	<0.001
SGLT2i (%)	20 (0.1)	16 (0.2)	0 (0)	3 (0.1)	0.601	0.254
GLP-1 RAs (%)	24 (0.1)	17 (0.2)	5 (0.2)	2 (0.1)	0.279	0.274
Insulin (%)	2556 (16.0)	1114 (10.7)	905 (33.4)	537 (18.6)	<0.001	<0.001
Blood pressure lowering drugs (excluding RASi) (%)	8340 (52.2)	4256 (41.0)	2238 (82.7)	1846 (64.0)	<0.001	<0.001
RASi (including ACEi and ARB) (%)	7004 (43.9)	3655 (35.2)	1747 (64.5)	1602 (55.5)	<0.001	<0.001
ACEi (%)	5189 (32.5)	2719 (26.2)	1226 (45.3)	1244 (43.1)	<0.001	0.103
ARB (%)	1846 (11.6)	943 (9.1)	538 (19.9)	365 (12.7)	<0.001	<0.001
Lipid-lowering drugs (excluding statins) (%)	676 (4.2)	388 (3.7)	147 (5.4)	142 (4.9)	0.004	0.391
Statins (%)	6945 (43.5)	4043 (39.0)	1546 (57.1)	1356 (47.0)	<0.001	<0.001
Aspirin (%)	3431 (21.5)	1623 (15.6)	1025 (37.9)	783 (27.1)	<0.001	<0.001
**Medical history**						
Sensory neuropathy (%)	770 (4.8)	288 (2.8.0)	288 (10.6)	194 (6.7)	<0.001	<0.001
Diabetic retinopathy (%)	3920 (24.5)	1928 (18.6)	999 (36.9)	992 (34.4)	<0.001	0.048
Prior severe hypoglycaemia (%)	1383 (8.7)	598 (5.8)	483 (17.8)	302 (10.5)	<0.001	<0.001
Prior cardiovascular disease (%)	3473 (21.7)	1577 (15.2)	1077 (39.8)	819 (28.4)	<0.001	<0.001
Prior cancer (%)	902 (5.7)	474 (4.6)	220 (8.1)	208 (7.2)	<0.001	0.197

	Overall	Without CKD at baseline and during follow-up	Pre-existing CKD at baseline	Incident CKD during follow-up	*P* value	
					Without CKD vs. Incident CKD	Pre-existing CKD vs. Incident CKD
ECI score (Range: <0 – 30) (%)						
No comorbidity (0)	12901 (80.8)	8945 (86.2)	1706 (63.0)	2250 (78.0)	<0.001	<0.001
Moderate ECI (<0 or 1-5)	1018 (6.4)	603 (5.8)	217 (8.0)	198 (6.9)		
High ECI (> 5)	2055 (12.9)	832 (8.0)	785 (29.0)	438 (15.2)		
**EQ-5D-3L**						
Mobility (%)						
No problems	14870 (93.1)	9962 (96.0)	2280 (84.2)	2628 (91.1)	<0.001	<0.001
Some or severe problems	1104 (6.9)	418 (4.0)	428 (15.8)	258 (8.9)		
Self-care (%)						
No problems	15590 (97.6)	10233 (98.6)	2552 (94.2)	2805 (97.2)	<0.001	<0.001
Some or severe problems	384 (2.4)	147 (1.4)	156 (5.8)	81 (2.8)		
Usual activities (%)						
No problems	15198 (95.1)	10059 (96.9)	2428 (89.7)	2711 (93.9)	<0.001	<0.001
Some or severe problems	776 (4.9)	321 (3.1)	280 (10.3)	175 (6.1)		
Pain/discomfort (%)						
No problems	11936 (74.7)	7894 (76.0)	1934 (71.4)	2108 (73.0)	<0.001	0.175
Some or severe problems	4038 (25.3)	2486 (24.0)	774 (28.6)	778 (27.0)		
Anxiety/depression (%)						
No problems	12869 (80.6)	8363 (80.6)	2183 (80.6)	2323 (80.5)	0.927	0.909
Some or severe problems	3105 (19.4)	2017 (19.4)	525 (19.4)	563 (19.5)		

Different groups were compared using ANOVA, Chi-square tests, and Fisher's exact tests as appropriate. ACEi, Angiotensin-converting enzyme inhibitors; ARB, Angiotensin II receptor blockers; BMI, Body mass index; eGFR, Estimated glomerular filtration rate; ECI, Elixhauser Comorbidity Index; EQ-5D-3L, EuroQol five-dimensional three-level questionnaire; GLP-1 RAs, Glucagon-like peptide-1 receptor agonists; RASi, Renin-angiotensin system inhibitors; SD, Standard deviation; SGLT2i, Sodium-glucose transporter 2 inhibitors; UACR, Urine albumin/creatinine ratio.

ESM Table 7. Diabetes-related complications, all-cause mortality and healthcare costs of individuals with T2D with pre-existing or incident CKD

	Pre-existing CKD	Incident CKD	*P* value
**Diabetes-related complications and all-cause mortality (n [%])**			
N	2708	2886	
Severe hypoglycaemia	462 (17.0)	374 (13.0)	<0.001
Cardiovascular disease	670 (24.7)	594 (20.6)	<0.001
Cancer	234 (8.6)	341 (11.8)	<0.001
End-stage kidney disease	823 (30.4)	172 (6.0)	<0.001
Death	950 (35.1)	470 (16.3)	<0.001
**Per-patient-per-year (PPPY) healthcare costs (US$) (mean [SD])**			
N	19262	24819	
Total healthcare costs	6987 (15835)	4395 (11947)	<0.001
Annual outpatient department costs	1266 (1598)	1086 (1492)	<0.001
Acute costs	3929 (10773)	2157 (6790)	<0.001
High-dependency unit costs	110 (1672)	83 (1576)	0.078
Intensive care unit costs	218 (3089)	163 (2734)	0.048
Rehabilitation and infirmary costs	1282 (6047)	789 (5399)	<0.001
Psychiatric costs	38 (1033)	47 (1301)	0.441
Accident and emergency costs	89 (197)	58 (154)	<0.001

CKD, Chronic kidney disease; SD, Standard deviation.

ESM Table 8. Fit statistics for latent class models from two to six classes

Number of classes	N	Likelihood	BIC	Class population shares (%)					
				Class 1	Class 2	Class 3	Class 4	Class 5	Class 6
2	2886	-30805.32	61985.13	1117 (38.70)	1769 (61.30)				
3	2886	-30470.19	61506.08	900 (31.19)	1031 (35.72)	955 (33.09)			
4	2886	-30334.97	61426.86	529 (18.33)	612 (21.21)	979 (33.92)	766 (26.54)		
5	2886	-30250.21	61448.57	994 (34.44)	706 (24.46)	574 (19.89)	451 (15.63)	161 (5.58)	
6	2886	-30169.17	61477.72	697 (24.15)	628 (21.76)	580 (20.10)	429 (14.86)	380 (13.17)	172 (5.96)

BIC, Bayesian information criterion.

ESM Table 9. Definitions and derivation of terminologies used for naming of latent classes

Terminology	Definition and derivation of terminology
Age at diagnosis (years)	**The mean and median age at diagnosis of T2D.**
Young-onset<br>Median (range): 44 (37-51) years for Class 1	Among the derived classes, the mean±SD age at diagnosis of T2D was 44.4±10.3 years, and the median (range) was 44 (37-51) years.
Middle-age onset<br>Median (range): 55 (48-60) years for Class 3; 54 (49-59) years for Class 4	Among the derived classes, the mean±SD age at diagnosis of T2D was 54.2±10.0 years and 54.1±7.6 years, and the median (range) was 55 (48-60) and 54 (49-59) years, respectively.
Old-age onset<br>Median (range): 66 (62-71) years for Class 2	Among the derived classes, the mean±SD age at diagnosis of T2D was 66.9±6.9 years, and the median (range) was 66 (62-71) years.
Comorbidities	**Severity of the participant's comorbidities. Defined by ECI score (<0–30): An ECI score of 0 is considered no comorbidity; an ECI score <0 or between 1–5 is considered moderate ECI; an ECI score >5 is considered high ECI.**
Few comorbidities	Among the derived classes, 14.0% had moderate or high ECI.
Moderate comorbidities	Among the derived classes, 25.3%-27.8% had moderate or high ECI.
Medications	**The participant's medication status. Defined by the proportion of participants treated with at least three out of the six medications used in the LCA: Glucose-lowering drugs, Insulin, Blood pressure lowering drugs (excluding RASi), RASi, Statins, and Aspirin.**
Few medications	Among the derived classes, the proportion of participants using at least three out of the six medications used for LCA was 15.6%.
Multiple medications	Among the derived classes, the proportions of participants using at least three out of the six medications used for LCA were between 70.7% and 98.9%.

ECI, Elixhauser Comorbidity Index; LCA, Latent class analysis; RASi, Renin-angiotensin system inhibitors; SD, standard deviation; T2D, Type 2 diabetes.

ESM Table 10. Covariates associated with class membership identified using multinomial logistic regression relative to Class 2

	Class 1 vs. Class 2		Class 3 vs. Class 2		Class 4 vs. Class 2	
	**aOR**	**95% CI**	**aOR**	**95% CI**	**aOR**	**95% CI**
**Personal characteristics**						
Sex (ref: Female)						
Male	0.82	0.43-1.57	1.10	0.64-1.89	0.93	0.54-1.58
Age at assessment (years) (ref: < 60)						
≥ 60	0.08***	0.04-0.15	0.07***	0.04-0.13	0.12***	0.06-0.21
Education level (ref: Primary, illiterate or others)						
Middle or high school	0.96	0.61-1.52	1.00	0.69-1.44	1.04	0.73-1.50
College or above	1.07	0.50-2.28	0.66	0.34-1.28	0.97	0.51-1.84
Occupation status (ref: Employed (full-time/part-time))						
Housewife/students/unemployed	0.54	0.26-1.13	0.61	0.33-1.15	0.70	0.37-1.33
Retired	0.78	0.42-1.44	0.61	0.36-1.03	0.74	0.44-1.25
Family history of diabetes (ref: False)	2.53***	1.64-3.91	1.36	0.96-1.93	1.68**	1.19-2.37
**Lifestyle factors**						
Consumption of alcohol (ref: Never)						
Ex-drinker	0.71	0.37-1.34	0.57*	0.33-1.00	0.91	0.55-1.50
Current drinker	0.80	0.48-1.35	0.83	0.54-1.27	0.90	0.59-1.37
Smoking (ref: Never)						
Ex-smoker	1.07	0.60-1.92	0.98	0.61-1.59	1.11	0.70-1.75
Current smoker	0.63	0.30-1.30	0.51*	0.27-0.95	0.66	0.36-1.21
**Clinical and biochemical characteristics**						
Central obesity (ref: Normal)	0.99	0.57-1.72	0.89	0.57-1.37	1.29	0.83-2.01
BMI (kg/m^2) (ref: < 25)						
25-30	0.87	0.50-1.52	0.64*	0.41-1.00	0.77	0.50-1.20
≥ 30	1.00	0.49-2.07	0.53*	0.28-1.00	0.79	0.44-1.41
Fasting plasma glucose (mmol/l) (ref: < 7)						
≥ 7	4.31***	2.79-6.65	2.79***	1.98-3.94	2.71***	1.94-3.79
Diastolic blood pressure (mmHg) (ref: < 75)						
75-85	0.91	0.55-1.53	0.88	0.58-1.34	1.09	0.72-1.63
≥ 85	0.61	0.33-1.12	0.70	0.42-1.16	0.88	0.54-1.44

	Class 1 vs. Class 2		Class 3 vs. Class 2		Class 4 vs. Class 2	
	aOR	95% CI	aOR	95% CI	aOR	95% CI
Systolic blood pressure (mmHg) (ref: < 125)						
125-140	1.06	0.57-1.95	0.99	0.61-1.60	1.18	0.73-1.90
≥ 140	1.29	0.68-2.44	1.11	0.67-1.86	0.94	0.57-1.57
Total cholesterol (mmol/l) (ref: < 5.2)						
≥ 5.2	0.70	0.37-1.31	1.07	0.67-1.71	0.84	0.50-1.42
Triglycerides (mmol/l) (ref: < 1.69)						
1.69-2.26	0.92	0.52-1.63	0.86	0.54-1.36	0.90	0.57-1.41
≥ 2.26	0.98	0.55-1.76	0.92	0.56-1.52	0.73	0.45-1.19
High-density lipoprotein (HDL) cholesterol (mmol/l) (ref: < 1.3)						
1.3-1.55	0.61	0.37-1.01	0.64*	0.43-0.96	0.71	0.48-1.05
≥ 1.55	0.88	0.48-1.63	0.88	0.54-1.44	0.85	0.52-1.39
Low-density lipoprotein (LDL) cholesterol (mmol/l) (ref: < 1.4)						
1.4-1.8	0.81	0.33-1.96	0.73	0.26-2.04	1.56	0.76-3.19
1.8-2.6	0.61	0.28-1.35	1.21	0.51-2.90	1.07	0.55-2.06
≥ 2.6	0.62	0.27-1.45	1.99	0.82-4.83	1.02	0.51-2.04
Haemoglobin (g/l) (ref: Normal)						
Anaemia	1.36	0.82-2.26	1.66*	1.08-2.54	1.51*	1.01-2.27
Plasma albumin (g/l) (ref: 35-50)						
< 35	1.14	0.20-6.34	0.49	0.08-2.88	0.37	0.05-2.54
≥ 50	0.82	0.15-4.45	0.61	0.15-2.45	0.75	0.19-3.00
Alanine aminotransferase (mmol/l) (ref: < 10)						
10-40	0.75	0.21-2.63	1.12	0.35-3.63	1.31	0.41-4.17
≥ 40	0.72	0.18-2.82	1.24	0.35-4.40	1.51	0.44-5.21
Alkaline phosphatase (mmol/l) (ref: 44-147)						
< 44	0.74	0.29-1.88	0.93	0.43-1.99	1.03	0.53-2.00
≥ 147	1.43	0.30-6.88	0.56	0.14-2.15	0.83	0.18-3.79
Bilirubin (μmol/l) (ref: 5-21)						
< 5	2.06	0.43-10.01	1.51	0.32-7.10	3.13	0.75-13.05
≥ 21	0.56	0.20-1.56	0.92	0.46-1.85	0.55	0.24-1.25
eGFR (ml/min/1.73m^2) (ref: ≥ 90)						
60-90	0.38**	0.21-0.70	0.46**	0.27-0.79	0.58	0.33-1.03

	Class 1 vs. Class 2		Class 3 vs. Class 2		Class 4 vs. Class 2	
	aOR	95% CI	aOR	95% CI	aOR	95% CI
**Medical treatments**						
ACEi (ref: No)	3.19***	1.99-5.12	0.41***	0.28-0.60	3.20***	2.21-4.65
ARB (ref: No)	2.70**	1.39-5.22	0.26***	0.13-0.50	3.60***	2.16-5.97
Lipid-lowering drugs (excluding statins) (ref: No)	2.00	0.75-5.28	1.71	0.69-4.22	1.11	0.46-2.72
**Medical history**						
Sensory neuropathy (ref: No)	1.57	0.73-3.35	1.57	0.77-3.19	0.78	0.38-1.63
Diabetic retinopathy (ref: No)	3.08***	2.00-4.72	1.46*	1.01-2.10	1.24	0.86-1.79
Prior severe hypoglycaemia (ref: No)	7.80***	4.07-14.95	3.10***	1.59-6.03	2.35**	1.26-4.38
Prior cardiovascular disease (ref: No)	0.49**	0.31-0.78	0.11***	0.07-0.18	1.37	0.97-1.93
Prior cancer (ref: No)	1.31	0.62-2.74	0.90	0.48-1.68	1.08	0.59-1.97
**EQ-5D-3L**						
Mobility (ref: No problems)						
Some or severe problems	1.19	0.49-2.88	1.53	0.74-3.13	0.89	0.44-1.81
Self-care (ref: No problems)						
Some or severe problems	0.98	0.22-4.41	3.83*	1.09-13.53	1.43	0.43-4.78
Usual activities (ref: No problems)						
Some or severe problems	0.72	0.24-2.18	0.31*	0.11-0.89	0.57	0.23-1.41
Pain/discomfort (ref: No problems)						
Some or severe problems	0.91	0.56-1.47	0.90	0.60-1.33	0.93	0.63-1.37
Anxiety/depression (ref: No problems)						
Some or severe problems	0.97	0.56-1.67	1.03	0.66-1.62	1.01	0.65-1.57

aOR, Adjusted odds ratio; ACEi, Angiotensin-converting enzyme inhibitors; ARB, Angiotensin II receptor blockers; BMI, Body mass index; CI, Confidence interval; eGFR, Estimated glomerular filtration rate; ECI, Elixhauser Comorbidity Index; EQ-5D-3L, EuroQol five-dimensional three-level questionnaire; RASi, Renin-angiotensin system inhibitors; SD, Standard deviation. ****p*<0.001; ***p*<0.01; **p*<0.05.

ESM Table 11. Longitudinal estimates of mean per-patient-per-year (PPPY) healthcare costs (US$) using hierarchical generalised linear mixed models

Variables	Total healthcare costs	OPD costs	Acute care costs	HDU costs	ICU costs	Rehabilitation and infirmary costs	Psychiatric costs	Accident and emergency costs
LCA class (ref: Class 2)								
Class 1	1.43 (1.26-1.62) ***	1.41 (1.31-1.52) ***	1.25 (1.11-1.42) ***	1.18 (0.83-1.68)	1.54 (1.04-2.29) *	1.12 (0.93-1.34)	3.30 (1.28-8.47) *	1.08 (1.01-1.15) *
Class 3	1.17 (1.04-1.30) **	1.16 (1.09-1.24) ***	1.06 (0.95-1.18)	0.90 (0.64-1.26)	1.37 (0.94-1.99)	1.02 (0.87-1.21)	7.41 (3.37-16.27) ***	1.06 (0.99-1.12)
Class 4	1.00 (0.89-1.11)	1.07 (1.00-1.14)	0.96 (0.86-1.07)	1.15 (0.83-1.60)	1.55 (1.08-2.23) *	0.99 (0.84-1.17)	4.72 (1.83-12.23) **	1.00 (0.94-1.06)
Consumption of alcohol (ref: Never)								
Ex-drinker	1.25 (1.11-1.39) ***	1.08 (1.01-1.16) *	1.25 (1.13-1.39) ***	0.92 (0.67-1.27)	1.24 (0.88-1.75)	1.05 (0.90-1.22)	-	1.10 (1.04-1.17) ***
Current drinker	0.92 (0.84-1.01)	0.96 (0.91-1.01)	0.98 (0.90-1.07)	1.08 (0.84-1.38)	1.22 (0.94-1.59)	1.06 (0.91-1.22)	-	1.01 (0.96-1.06)
Family history of diabetes (ref: False)	0.88 (0.80-0.95) **	1.03 (0.98-1.08)	0.88 (0.81-0.96) **	0.97 (0.76-1.24)	0.91 (0.71-1.17)	1.00 (0.88-1.14)	0.98 (0.53-1.82)	0.98 (0.94-1.03)
Diastolic blood pressure (mmHg) (ref: < 75)								
75-85	0.91 (0.83-0.99) *	0.98 (0.93-1.03)	0.91 (0.83-0.99) *	0.82 (0.63-1.06)	1.03 (0.78-1.36)	0.96 (0.84-1.09)	0.39 (0.20-0.74) **	0.99 (0.95-1.04)
≥ 85	0.93 (0.85-1.02)	1.00 (0.95-1.06)	1.03 (0.94-1.13)	0.95 (0.71-1.26)	0.89 (0.67-1.19)	0.92 (0.80-1.06)	0.63 (0.33-1.21)	1.02 (0.97-1.07)
Plasma albumin (g/l) (ref: 35-50)								
< 35	3.09 (2.19-4.35) ***	1.52 (1.23-1.88) ***	2.12 (1.59-2.84) ***	4.65 (1.32-16.43) *	1.13 (0.54-2.35)	1.29 (0.90-1.84)	-	1.35 (1.17-1.57) ***
≥ 50	0.66 (0.48-0.91) *	0.85 (0.70-1.03)	0.88 (0.62-1.23)	1.45 (0.21-10.00)	0.61 (0.13-2.83)	0.52 (0.28-0.96) *	-	0.91 (0.75-1.10)
Alkaline phosphatase (mmol/l) (ref: 44-147)								
< 44	0.93 (0.79-1.10)	0.95 (0.86-1.05)	0.92 (0.78-1.08)	1.08 (0.68-1.72)	1.01 (0.55-1.85)	0.94 (0.69-1.27)	-	1.02 (0.93-1.13)
≥ 147	1.54 (1.12-2.12) **	1.08 (0.89-1.31)	1.33 (1.00-1.79)	1.68 (0.68-4.14)	1.33 (0.61-2.92)	0.94 (0.63-1.41)	-	1.06 (0.91-1.23)
eGFR (ml/min/1.73m^2) (ref: ≥ 90)								
60-90	0.78 (0.71-0.87) ***	0.84 (0.79-0.89) ***	0.85 (0.77-0.93) ***	0.95 (0.72-1.26)	1.13 (0.86-1.48)	1.07 (0.92-1.24)	1.00 (0.48-2.05)	0.97 (0.92-1.02)
Prior severe hypoglycaemia (ref: No)	1.58 (1.40-1.79) ***	1.25 (1.16-1.35) ***	1.49 (1.32-1.68) ***	1.00 (0.72-1.40)	1.04 (0.71-1.51)	1.03 (0.88-1.21)	0.68 (0.30-1.54)	1.20 (1.13-1.28) ***
Prior cardiovascular disease (ref: No)	1.24 (1.13-1.35) ***	1.08 (1.02-1.14) **	1.17 (1.07-1.27) ***	0.93 (0.72-1.21)	1.08 (0.81-1.42)	1.00 (0.88-1.14)	1.75 (0.90-3.40)	1.08 (1.03-1.14) **

Variables	Total healthcare costs	OPD costs	Acute care costs	HDU costs	ICU costs	Rehabilitation and infirmary costs	Psychiatric costs	Accident and emergency costs
Prior cancer (ref: No)	1.47 (1.27-1.70) ***	1.22 (1.12-1.33) ***	1.12 (0.98-1.29)	1.08 (0.72-1.61)	1.13 (0.72-1.78)	1.19 (0.99-1.44)	3.66 (1.43-9.40) **	1.12 (1.04-1.21) **
Mobility (ref: No problems)								
Some or severe problems	1.57 (1.33-1.86) ***	1.14 (1.03-1.26) *	1.40 (1.20-1.64) ***	1.28 (0.72-2.27)	1.12 (0.67-1.87)	1.03 (0.83-1.26)	0.48 (0.16-1.42)	1.08 (0.99-1.17)
Self-care (ref: No problems)								
Some or severe problems	1.31 (0.99-1.74)	0.96 (0.81-1.14)	1.10 (0.85-1.43)	0.89 (0.27-2.98)	1.60 (0.39-6.63)	1.11 (0.82-1.49)	0.45 (0.08-2.40)	1.11 (0.97-1.27)
Usual activities (ref: No problems)								
Some or severe problems	1.05 (0.85-1.32)	1.22 (1.07-1.39) **	0.98 (0.80-1.21)	0.90 (0.42-1.90)	0.72 (0.31-1.65)	0.98 (0.74-1.30)	4.37 (1.02-18.66) *	1.03 (0.92-1.15)
Pain/discomfort (ref: No problems)								
Some or severe problems	1.05 (0.96-1.15)	1.06 (1.00-1.12) *	0.95 (0.87-1.04)	0.91 (0.69-1.20)	0.96 (0.71-1.29)	1.02 (0.90-1.16)	1.49 (0.80-2.78)	0.96 (0.91-1.01)
Anxiety/depression (ref: No problems)								
Some or severe problems	1.06 (0.95-1.17)	1.06 (0.99-1.12)	0.98 (0.89-1.08)	1.07 (0.80-1.43)	0.88 (0.63-1.22)	1.03 (0.90-1.19)	1.00 (0.53-1.87)	0.99 (0.94-1.04)
Year	1.07 (1.06-1.08) ***	1.01 (1.00-1.01) ***	1.10 (1.09-1.11) ***	1.01 (0.97-1.04)	1.02 (0.98-1.07)	1.01 (0.99-1.03)	1.07 (0.98-1.16)	1.03 (1.02-1.03) ***

Reported results are adjusted cost ratio (95% confidence intervals). Models were adjusted for the alcohol use, family history of diabetes, diastolic blood pressure, plasma albumin, alkaline phosphatase, eGFR, prior severe hypoglycaemia, prior cardiovascular disease, prior cancer, and EQ-5D-3L (mobility, self-care, usual activities, pain/discomfort, and anxiety/depression). Psychiatric cost exhibits high zero-inflation; the model was simplified by including a year-varying random slope only at the individual level, while adjusting for the variables mentioned earlier, except for alcohol use, plasma albumin and alkaline phosphatase. eGFR, Estimated glomerular filtration rate; EQ-5D-3L, EuroQol five-dimensional three-level questionnaire; HDU, High-dependency unit; ICU, Intensive care unit; LCA, Latent class analysis; OPD, Outpatient department. ****p*<0.001; ***p*<0.01; **p*<0.05.

ESM Table 12. Sensitivity analyses of longitudinal estimates of mean per-patient-per-year (PPPY) healthcare costs (US$) using hierarchical generalised linear mixed models based on winsorised costs

Variables	Total healthcare costs	OPD costs	Acute care costs	HDU costs	ICU costs	Rehabilitation and infirmary costs	Psychiatric costs	Accident and emergency costs
LCA class (ref: Class 2)								
Class 1	1.43 (1.26-1.62) ***	1.41 (1.31-1.52) ***	1.25 (1.11-1.41) ***	1.15 (0.85-1.56)	1.52 (1.07-2.16) *	1.10 (0.92-1.31)	2.21 (0.96-5.06)	1.08 (1.01-1.15) *
Class 3	1.17 (1.04-1.30) **	1.16 (1.09-1.24) ***	1.06 (0.95-1.18)	0.88 (0.65-1.19)	1.31 (0.94-1.82)	1.02 (0.87-1.19)	3.03 (1.52-6.05) **	1.06 (1.00-1.12)
Class 4	0.99 (0.89-1.11)	1.07 (1.00-1.14)	0.96 (0.86-1.07)	1.09 (0.82-1.45)	1.48 (1.07-2.04) *	0.99 (0.84-1.16)	3.13 (1.37-7.15) **	1.00 (0.94-1.06)
Consumption of alcohol (ref: Never)								
Ex-drinker	1.25 (1.12-1.39) ***	1.08 (1.01-1.16) *	1.25 (1.13-1.39) ***	0.92 (0.70-1.21)	1.19 (0.88-1.61)	1.04 (0.90-1.21)	-	1.10 (1.04-1.17) ***
Current drinker	0.92 (0.84-1.01)	0.96 (0.91-1.01)	0.98 (0.90-1.07)	1.06 (0.85-1.31)	1.20 (0.95-1.51)	1.05 (0.92-1.21)	-	1.01 (0.96-1.06)
Family history of diabetes (ref: False)	0.88 (0.80-0.96) **	1.03 (0.98-1.08)	0.88 (0.81-0.96) **	0.95 (0.76-1.18)	0.92 (0.73-1.15)	1.00 (0.89-1.13)	1.06 (0.65-1.74)	0.98 (0.94-1.03)
Diastolic blood pressure (mmHg) (ref: < 75)								
75-85	0.91 (0.83-0.99) *	0.98 (0.93-1.03)	0.91 (0.83-0.99) *	0.87 (0.70-1.09)	1.02 (0.80-1.30)	0.97 (0.86-1.10)	0.58 (0.32-1.03)	0.99 (0.94-1.04)
≥ 85	0.93 (0.85-1.03)	1.00 (0.95-1.06)	1.03 (0.94-1.13)	0.98 (0.77-1.26)	0.87 (0.68-1.12)	0.93 (0.81-1.07)	0.71 (0.39-1.27)	1.02 (0.97-1.07)
Plasma albumin (g/l) (ref: 35-50)								
< 35	3.08 (2.19-4.33) ***	1.52 (1.23-1.88) ***	2.12 (1.59-2.83) ***	3.02 (1.01-9.01) *	1.13 (0.59-2.15)	1.28 (0.91-1.80)	-	1.34 (1.16-1.55) ***
≥ 50	0.66 (0.48-0.91) *	0.85 (0.70-1.03)	0.88 (0.62-1.23)	1.36 (0.26-7.27)	0.59 (0.15-2.34)	0.53 (0.30-0.95) *	-	0.91 (0.75-1.10)
Alkaline phosphatase (mmol/l) (ref: 44-147)								
< 44	0.93 (0.79-1.10)	0.95 (0.86-1.05)	0.92 (0.78-1.08)	1.06 (0.71-1.61)	0.96 (0.56-1.64)	0.92 (0.69-1.22)	-	1.03 (0.93-1.13)
≥ 147	1.53 (1.11-2.10) **	1.09 (0.90-1.32)	1.33 (0.99-1.78)	1.40 (0.64-3.07)	1.15 (0.59-2.24)	0.94 (0.64-1.38)	-	1.06 (0.92-1.23)
eGFR (ml/min/1.73m^2) (ref: ≥ 90)								
60-90	0.78 (0.71-0.87) ***	0.84 (0.79-0.90) ***	0.85 (0.77-0.93) ***	1.00 (0.79-1.27)	1.08 (0.85-1.37)	1.07 (0.93-1.23)	0.80 (0.44-1.44)	0.97 (0.92-1.02)
Prior severe hypoglycaemia (ref: No)	1.58 (1.39-1.79) ***	1.25 (1.16-1.35) ***	1.49 (1.32-1.68) ***	1.04 (0.78-1.39)	0.99 (0.72-1.38)	1.03 (0.88-1.19)	0.58 (0.30-1.14)	1.19 (1.12-1.27) ***
Prior cardiovascular disease (ref: No)	1.24 (1.13-1.35) ***	1.08 (1.02-1.14) **	1.17 (1.07-1.27) ***	0.96 (0.77-1.21)	1.00 (0.78-1.28)	1.01 (0.89-1.14)	1.43 (0.82-2.49)	1.08 (1.03-1.13) **

Variables	Total healthcare costs	OPD costs	Acute care costs	HDU costs	ICU costs	Rehabilitation and infirmary costs	Psychiatric costs	Accident and emergency costs
Prior cancer (ref: No)	1.47 (1.27-1.70) ***	1.22 (1.12-1.33) ***	1.12 (0.98-1.29)	1.06 (0.75-1.50)	1.03 (0.70-1.53)	1.14 (0.95-1.36)	2.17 (0.97-4.86)	1.12 (1.04-1.21) **
Mobility (ref: No problems)								
Some or severe problems	1.57 (1.33-1.86) ***	1.14 (1.03-1.26) *	1.40 (1.20-1.64) ***	1.33 (0.81-2.19)	1.18 (0.75-1.87)	1.00 (0.82-1.22)	0.81 (0.35-1.89)	1.08 (0.99-1.17)
Self-care (ref: No problems)								
Some or severe problems	1.31 (0.99-1.74)	0.96 (0.81-1.14)	1.11 (0.85-1.43)	0.77 (0.27-2.20)	1.58 (0.48-5.16)	1.09 (0.82-1.46)	0.77 (0.19-3.14)	1.11 (0.97-1.26)
Usual activities (ref: No problems)								
Some or severe problems	1.06 (0.85-1.32)	1.22 (1.07-1.39) **	0.98 (0.80-1.21)	0.91 (0.47-1.74)	0.69 (0.33-1.45)	1.01 (0.77-1.32)	1.58 (0.53-4.68)	1.03 (0.92-1.15)
Pain/discomfort (ref: No problems)								
Some or severe problems	1.05 (0.96-1.15)	1.06 (1.00-1.12) *	0.95 (0.87-1.03)	0.95 (0.75-1.21)	0.99 (0.76-1.29)	1.02 (0.90-1.15)	1.32 (0.77-2.29)	0.96 (0.91-1.01)
Anxiety/depression (ref: No problems)								
Some or severe problems	1.05 (0.95-1.17)	1.06 (0.99-1.12)	0.98 (0.89-1.08)	1.10 (0.85-1.41)	0.88 (0.66-1.17)	1.02 (0.89-1.17)	0.85 (0.51-1.42)	0.99 (0.94-1.04)
Year	1.07 (1.06-1.08) ***	1.01 (1.00-1.01) ***	1.10 (1.09-1.11) ***	1.01 (0.98-1.04)	1.04 (1.00-1.08)	1.01 (0.99-1.03)	1.04 (0.96-1.12)	1.03 (1.02-1.03) ***

Reported results are adjusted cost ratio (95% confidence intervals). Models were adjusted for the alcohol use, family history of diabetes, diastolic blood pressure, plasma albumin, alkaline phosphatase, eGFR, prior severe hypoglycaemia, prior cardiovascular disease, prior cancer, and EQ-5D-3L (mobility, self-care, usual activities, pain/discomfort, and anxiety/depression) based on winsorised healthcare costs. Psychiatric cost exhibits high zero-inflation; the model was simplified by including a year-varying random slope only at the individual level, while adjusting for the variables mentioned earlier, except for alcohol use, plasma albumin and alkaline phosphatase. eGFR, Estimated glomerular filtration rate; EQ-5D-3L, EuroQol five-dimensional three-level questionnaire; HDU, High-dependency unit; ICU, Intensive care unit; LCA, Latent class analysis; OPD, Outpatient department. ***p<0.001; **p<0.01; *p<0.05.

ESM Table 13. Sensitivity analyses of longitudinal estimates of mean per-patient-per-year (PPPY) healthcare costs (US$) using hierarchical linear models based on original costs

Variables	Total healthcare costs	OPD costs	Acute care costs	HDU costs	ICU costs	Rehabilitation and infirmary costs	Psychiatric costs	Accident and emergency costs
**Fixed effects**								
Intercept	2532 (471) ***	796 (70) ***	1247 (268) ***	119 (47) *	112 (81)	205 (178)	54 (49)	27 (7) ***
LCA class (ref: Class 2)								
Class 1	1723 (383) ***	373 (57) ***	677 (217) **	49 (37)	202 (65) **	240 (146)	30 (40)	15 (6) **
Class 3	738 (337) *	167 (50) ***	270 (191)	-18 (32)	37 (56)	164 (128)	92 (35) **	4 (5)
Class 4	28 (338)	42 (50)	-174 (192)	44 (33)	125 (57) *	-44 (129)	15 (35)	0 (5)
Consumption of alcohol (ref: Never)								
Ex-drinker	1184 (339) ***	121 (51) *	860 (192) ***	29 (33)	75 (57)	100 (129)	-33 (35)	13 (5) **
Current drinker	-97 (266)	-49 (40)	-42 (151)	40 (25)	72 (44)	-77 (101)	-49 (27)	-5 (4)
Family history of diabetes (ref: False)	-697 (265) **	28 (38)	-373 (158) *	13 (23)	-73 (40)	-221 (96) *	-11 (25)	-10 (4) *
Diastolic blood pressure (mmHg) (ref: < 75)								
75-85	-693 (274) *	-41 (41)	-340 (157) *	-41 (26)	-30 (45)	-203 (103)	-30 (28)	-5 (4)
≥ 85	-331 (288)	19 (43)	-87 (166)	-32 (28)	-27 (48)	-176 (109)	11 (29)	-2 (4)
Plasma albumin (g/l) (ref: 35-50)								
< 35	8622 (1063) ***	555 (157) ***	5434 (603) ***	74 (110)	501 (191) **	1580 (404) ***	11 (113)	129 (15) ***
≥ 50	-1691 (952)	-121 (144)	-894 (538)	-63 (93)	-122 (162)	-406 (363)	-26 (100)	-21 (14)
Alkaline phosphatase (mmol/l) (ref: 44-147)								
< 44	-475 (495)	-27 (74)	-109 (279)	5 (47)	-6 (82)	-317 (188)	-37 (51)	-7 (7)
≥ 147	4208 (995) ***	89 (147)	2385 (562) ***	161 (97)	671 (169) ***	372 (379)	-45 (103)	23 (14)
eGFR (ml/min/1.73m^2) (ref: ≥ 90)								
60-90	-1016 (306) ***	-220 (46) ***	-584 (173) ***	-81 (29) **	-59 (51)	-26 (116)	-41 (31)	-8 (4)
Prior severe hypoglycaemia (ref: No)	3130 (386) ***	409 (58) ***	1953 (218) ***	14 (38)	91 (66)	581 (147) ***	-17 (40)	44 (6) ***
Prior cardiovascular disease (ref: No)	819 (277) **	75 (41)	411 (157) **	22 (27)	34 (47)	202 (105)	26 (29)	19 (4) ***
Prior cancer (ref: No)	1767 (447) ***	223 (67) ***	792 (253) **	16 (44)	49 (76)	649 (170) ***	16 (47)	19 (6) **
Mobility (ref: No problems)								
Some or severe problems	2087 (520) ***	193 (77) *	1256 (294) ***	-10 (51)	-8 (89)	532 (198) **	42 (54)	23 (7) **
Self-care (ref: No problems)								

Variables	Total healthcare costs	OPD costs	Acute care costs	HDU costs	ICU costs	Rehabilitation and infirmary costs	Psychiatric costs	Accident and emergency costs
Some or severe problems	2434 (874) **	-31 (130)	588 (494)	11 (89)	184 (154)	1546 (333) ***	-126 (92)	29 (13) *
Usual activities (ref: No problems)								
Some or severe problems	421 (679)	199 (101) *	126 (384)	-3 (68)	-76 (118)	50 (259)	138 (72)	10 (10)
Pain/discomfort (ref: No problems)								
Some or severe problems	223 (276)	108 (41) **	-56 (156)	-29 (27)	12 (46)	166 (105)	-22 (28)	-2 (4)
Anxiety/depression (ref: No problems)								
Some or severe problems	158 (305)	57 (46)	45 (173)	15 (29)	-25 (51)	-10 (116)	52 (31)	2 (4)
Year	541 (34) ***	55 (4) ***	315 (20) ***	3 (3)	5 (6)	160 (17) ***	0 (3)	9 (0) ***
**Random effects**								
Variance intercept (SD)	3695	726	2004	247	423	0	466	62
Variance year (SD)	1130	114	713	0	0	635	0	18

Reported results are estimates (standard error). Models were adjusted for the alcohol use, family history of diabetes, diastolic blood pressure, plasma albumin, alkaline phosphatase, eGFR, prior severe hypoglycaemia, prior cardiovascular disease, prior cancer, and EQ-5D-3L (mobility, self-care, usual activities, pain/discomfort, and anxiety/depression). eGFR, Estimated glomerular filtration rate; EQ-5D-3L, EuroQol five-dimensional three-level questionnaire; HDU, High-dependency unit; ICU, Intensive care unit; LCA, Latent class analysis; OPD, Outpatient department; SD, Standard deviation. ***p<0.001; **p<0.01; *p<0.05.

ESM Table 14. Sensitivity analyses of longitudinal estimates of mean per-patient-per-year (PPPY) healthcare costs (US$) using hierarchical linear models based on winsorised costs

Variables	Total healthcare costs	OPD costs	Acute care costs	HDU costs	ICU costs	Rehabilitation and infirmary costs	Psychiatric costs	Accident and emergency costs
**Fixed effects**								
Intercept	2473 (452) ***	775 (67) ***	1228 (262) ***	58 (23) *	99 (47) *	246 (163)	41 (22)	27 (6) ***
LCA class (ref: Class 2)								
Class 1	1688 (369) ***	360 (55) ***	660 (213) **	43 (18) *	153 (38) ***	268 (133) *	17 (18)	16 (5) **
Class 3	727 (324) *	169 (49) ***	265 (187)	-14 (16)	14 (33)	143 (117)	39 (16) *	5 (5)
Class 4	-39 (325)	42 (49)	-166 (188)	8 (16)	69 (33) *	-51 (118)	6 (16)	1 (5)
Consumption of alcohol (ref: Never)								
Ex-drinker	1181 (326) ***	110 (49) *	871 (188) ***	16 (16)	56 (34)	131 (118)	-10 (16)	13 (5) **
Current drinker	-132 (256)	-48 (38)	-46 (148)	12 (12)	52 (26) *	-91 (93)	-19 (12)	-4 (4)
Family history of diabetes (ref: False)	-689 (256) **	26 (36)	-360 (154) *	4 (12)	-50 (24) *	-242 (88) **	-3 (12)	-10 (4) **
Diastolic blood pressure (mmHg) (ref: < 75)								
75-85	-658 (263) *	-36 (39)	-333 (153) *	-12 (13)	-20 (27)	-190 (95) *	-8 (13)	-5 (4)
≥ 85	-280 (277)	18 (41)	-85 (162)	-6 (13)	-26 (28)	-197 (99) *	1 (13)	-2 (4)
Plasma albumin (g/l) (ref: 35-50)								
< 35	8373 (1020) ***	574 (151) ***	5425 (590) ***	16 (53)	294 (109) **	1666 (369) ***	46 (50)	125 (14) ***
≥ 50	-1658 (915)	-117 (139)	-881 (527)	-45 (45)	-99 (95)	-421 (332)	-15 (47)	-20 (13)
Alkaline phosphatase (mmol/l) (ref: 44-147)								
< 44	-454 (476)	-18 (71)	-101 (274)	6 (23)	-45 (49)	-240 (173)	-24 (23)	-6 (7)
≥ 147	3900 (956) ***	101 (142)	2375 (550) ***	107 (48) *	273 (99) **	346 (346)	-34 (46)	24 (13)
eGFR (ml/min/1.73m^2) (ref: ≥ 90)								
60-90	-949 (294) **	-199 (44) ***	-571 (170) ***	-37 (14) **	-56 (30)	-15 (106)	-30 (14) *	-8 (4) *
Prior severe hypoglycaemia (ref: No)	3039 (371) ***	387 (55) ***	1932 (214) ***	19 (19)	19 (39)	579 (134) ***	-1 (18)	42 (5) ***
Prior cardiovascular disease (ref: No)	778 (266) **	77 (40)	412 (153) **	36 (13) **	-2 (27)	228 (96) *	14 (13)	19 (4) ***
Prior cancer (ref: No)	1790 (430) ***	231 (64) ***	814 (248) **	23 (21)	23 (45)	695 (156) ***	13 (21)	19 (6) **
Mobility (ref: No problems)								
Some or severe problems	2054 (499) ***	199 (75) **	1186 (288) ***	-11 (25)	24 (52)	533 (181) **	16 (24)	24 (7) ***
Self-care (ref: No problems)								
Some or severe problems	2418 (839) **	-29 (125)	665 (484)	-8 (43)	138 (88)	1617 (304) ***	-27 (41)	29 (12) *

Variables	Total healthcare costs	OPD costs	Acute care costs	HDU costs	ICU costs	Rehabilitation and infirmary costs	Psychiatric costs	Accident and emergency costs
Usual activities (ref: No problems)								
Some or severe problems	459 (653)	201 (98) *	122 (376)	1 (33)	-63 (68)	38 (236)	34 (32)	10 (9)
Pain/discomfort (ref: No problems)								
Some or severe problems	217 (266)	110 (40) **	-59 (153)	-8 (13)	17 (27)	143 (96)	-13 (13)	-2 (4)
Anxiety/depression (ref: No problems)								
Some or severe problems	131 (293)	46 (44)	36 (169)	17 (14)	-32 (30)	-25 (106)	18 (14)	1 (4)
Year	547 (32) ***	55 (4) ***	313 (19) ***	4 (2) *	11 (4) **	134 (13) ***	-1 (1)	8 (0) ***
**Random effects**								
Variance intercept (SD)	3642	712	2027	59	0	1021	216	58
Variance year (SD)	1118	115	694	26	76	427	17	16

Reported results are estimates (standard error). Models were adjusted for the alcohol use, family history of diabetes, diastolic blood pressure, plasma albumin, alkaline phosphatase, eGFR, prior severe hypoglycaemia, prior cardiovascular disease, prior cancer, and EQ-5D-3L (mobility, self-care, usual activities, pain/discomfort, and anxiety/depression) based on winsorised healthcare costs. eGFR, Estimated glomerular filtration rate; EQ-5D-3L, EuroQol five-dimensional three-level questionnaire; HDU, High-dependency unit; ICU, Intensive care unit; LCA, Latent class analysis; OPD, Outpatient department; SD, Standard deviation. ***$p<0.001$; **$p<0.01$; *$p<0.05$.

ESM Fig. 1. Plots of mean and standard deviation of the imputed variables against iteration number for the imputed data

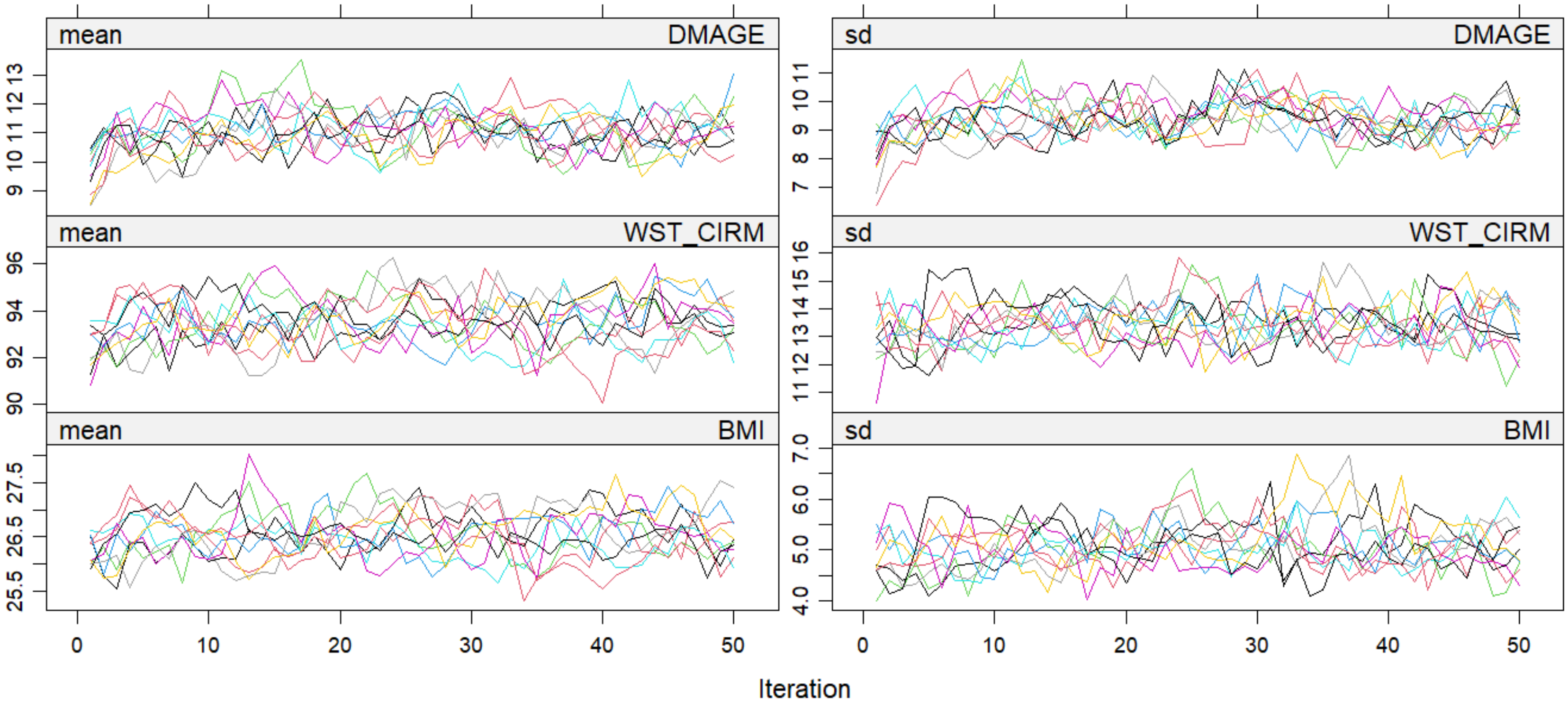

Each line represents 1 of the 10 imputed datasets. Convergence is indicated by the overlap of lines and lack of any trend. BMI, Body mass index; DMAGE, Duration of diabetes; SD, Standard deviation; WST_CIRM, Waist circumference.

ESM Fig. 2. Flow chart of the study

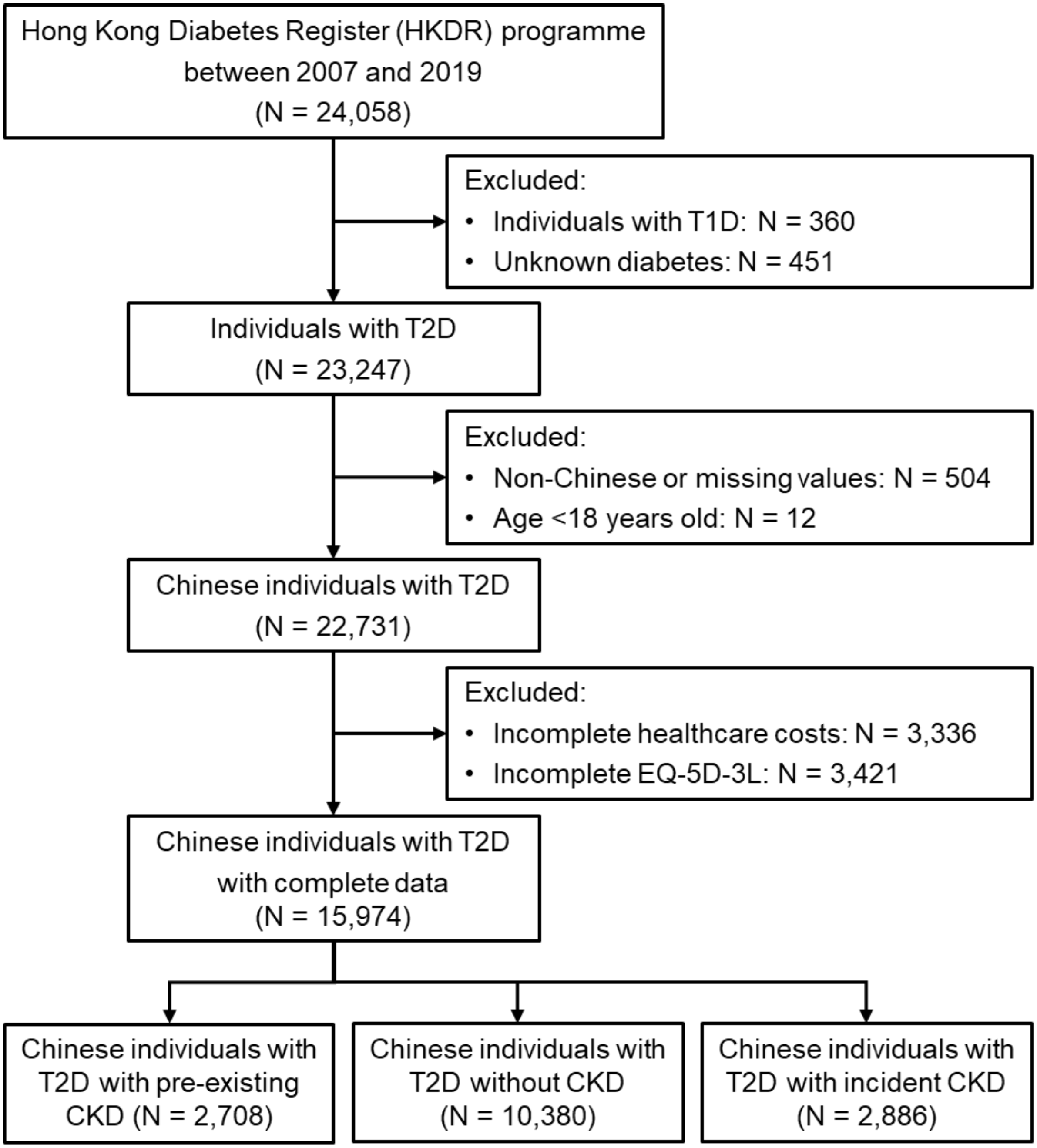

CKD, Chronic kidney disease; EQ-5D-3L, EuroQol five-dimensional three-level questionnaire; T1D, Type 1 diabetes; T2D, Type 2 diabetes.

ESM Fig. 3. Distribution of latent class indicators based on class assignment in the four-class model

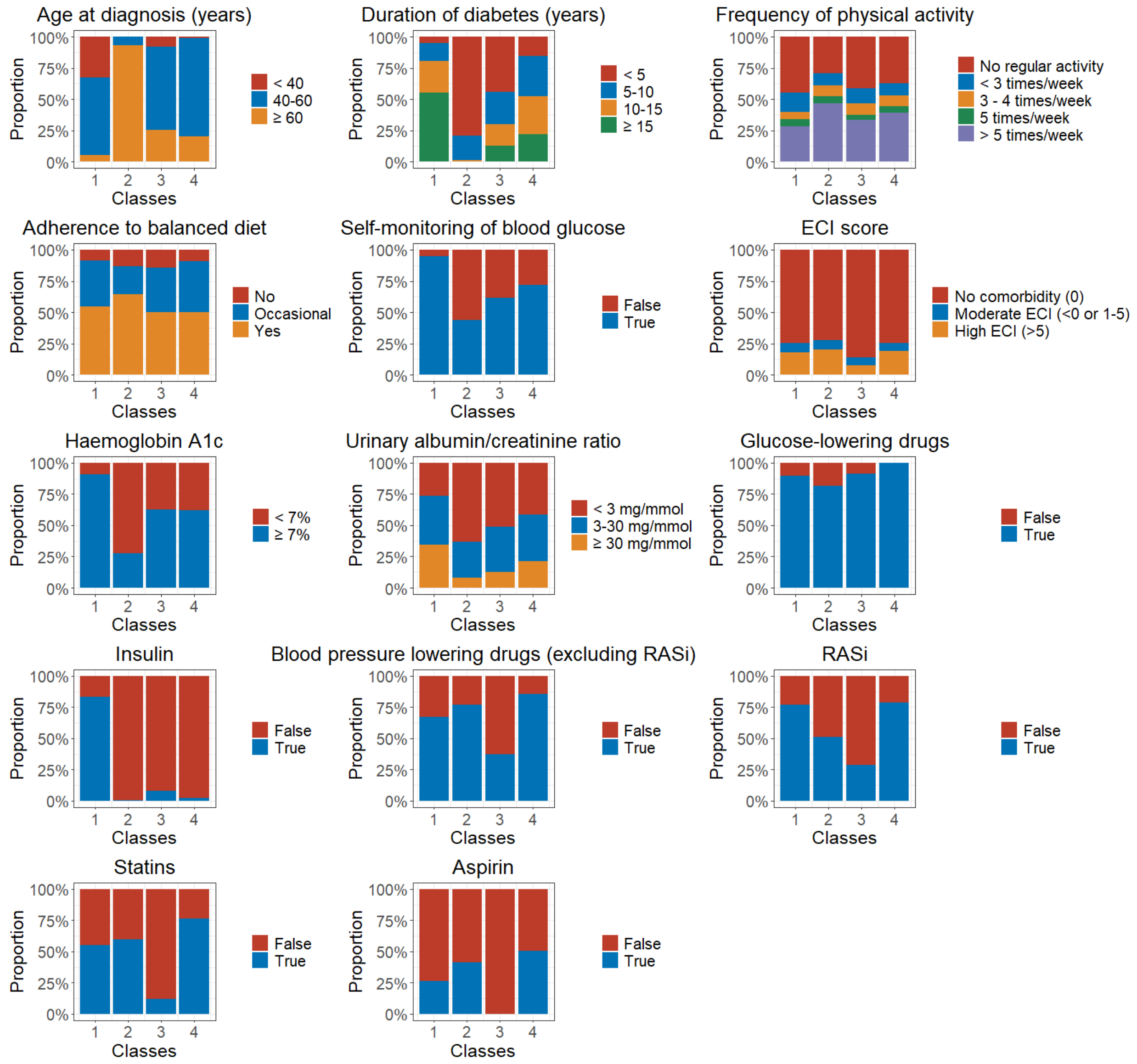

ECI, Elixhauser Comorbidity Index; RASi, Renin-angiotensin system inhibitors.
